# Identification and Functional Characterization of Chitinase Genes During Larva–Pupa–Adult Transitions in *Tuta absoluta*

**DOI:** 10.3390/insects17010114

**Published:** 2026-01-20

**Authors:** Kangkang Xu, Yue Wang, Shuyan Yan, Fanghao Wan, Guy Smagghe, Wenjia Yang

**Affiliations:** 1 Guizhou Key Laboratory of Agricultural Biosecurity, Key Laboratory of Surveillance and Management of Invasive Alien Species in Guizhou Province, College of Biological and Environmental Engineering, Guiyang University, Guiyang 550005, China; kkxu1988@163.com (K.X.); yanshuyan27@126.com (S.Y.); 2 Institute of Plant Protection, Chinese Academy of Agricultural Sciences, Beijing 100193, China; wanfanghao@caas.cn; 3 Institute of Entomology, Guizhou University, Guiyang 550025, China; guysma9@gmail.com; 4 Department of Biology, Vrije Universiteit Brussel (VUB), 1050 Brussels, Belgium

**Keywords:** *Tuta absoluta*, chitinase, molting and metamorphosis, RNA interference, carbon quantum dots, cuticle remodeling, developmental physiology, pest management strategies

## Abstract

Chitinases are crucial in the molting process of insects and represent potential targets for the development of RNA interference (RNAi)-based insecticidal strategies. In this study, we identified and characterized 11 chitinase genes (*TaChts*) in *T. absoluta*, each harboring at least one GH18 catalytic domain. Phylogenetic analysis placed these proteins into 11 established groups (I-X and h), revealing conserved lineage-specific patterns. Spatiotemporal expression profiling showed that most *TaChts* peak during key developmental transitions. *TaCht1*, *TaCht2*, *TaCht3*, *TaCht5*, *TaCht6*, *TaCht7*, *TaCht10*, *TaCht-h*, and *TaIDGF* were predominantly expressed in the integument, whereas *TaCht8* and *TaCht11* showed gut-enriched expression, indicating functional specialization. Stage-tailored RNAi assays demonstrated that silencing *TaCht5*, *TaCht7*, *TaCht10*, and *TaIDGF* caused pronounced mortality in both larvae and pupae. In larvae, delivery of dsRNA using carbon quantum dots (CQDs) disrupted molting: knockdown of *TaCht7*, *TaCht10*, and *TaIDGF* prevented shedding of the old cuticle, whereas *TaCht5* silencing produced severe cuticular shrinkage and blackening. Hematoxylin and eosin staining revealed detachment of epidermal cells from the cuticle, providing cytological evidence of disrupted cuticle–epidermis remodeling. In pupae, microinjection of dsRNA induced cuticular collapse, melanization, and eclosion failure due to persistent adhesion of the pupal case, accompanied by marked reductions in chitinase activity. Together, our findings highlight the critical roles of four key chitinases in endocuticular turnover and metamorphic progression in *T. absoluta*, offering mechanistic insight into chitin-mediated developmental processes. The identified genes represent strong candidates for RNAi-based, species-specific pest management strategies against this globally significant insect.

## Simple Summary

The South American tomato leaf miner, *Tuta absoluta*, is a highly destructive invasive pest of solanaceous crops worldwide. Intensive reliance on chemical insecticides has led to widespread resistance and heightened environmental concerns, underscoring the need for species-specific and physiologically informed alternatives. Chitinases, which mediate chitin turnover during molting and metamorphosis, represent promising molecular targets for RNA interference (RNAi)-based control strategies. Therefore, this study aims to systematically characterize the chitinase family genes in *T. absoluta* and investigate their roles in larva–pupa–adult transitions through molecular characterization, spatiotemporal expression, and RNAi analyses. These findings reveal that four key chitinases (*TaCht5*, *TaCht7*, *TaCht10*, and *TaIDGF*) are indispensable for the molting physiology and metamorphic progression of *T. absoluta*, thereby providing potential molecular targets for leaf miner pest management.

## 1. Introduction

The South American tomato leaf miner, *Tuta absoluta* (Meyrick) (Lepidoptera: Gelechiidae), is an invasive pest originating from Peru that has rapidly become one of the most destructive species affecting solanaceous crops worldwide [1,2,3]. Its larvae mine leaves, stems, and fruits across all plant growth stages, generating serpentine tunnels that directly compromise photosynthesis and yield while predisposing damaged tissues to secondary infection by pathogens [4]. Presently, management relies heavily on chemical insecticides; however, chronic and indiscriminate use has produced widespread resistance, ecological contamination, and pest resurgence [5,6]. These constraints highlight the urgent need for physiologically informed, species-specific, and environmentally compatible alternatives to conventional chemical control.

RNA interference (RNAi) has emerged as a promising molecular tool for pest management due to its capacity to induce post-transcriptional gene silencing through exogenous double-stranded RNA (dsRNA), thereby disrupting essential physiological processes and causing aberrant development or mortality [7,8,9,10]. For insect physiologists, RNAi also provides an unparalleled experimental approach for resolving gene function and dissecting the molecular underpinnings of development, endocrinology, cuticle physiology, and metamorphic transitions. RNAi exhibits high specificity and efficiency in its mechanism of action; however, the effective delivery of dsRNA to target sites remains a significant challenge in practical applications. To address this issue, the utilization of nanomaterials as delivery carriers presents a promising strategy to overcome critical obstacles, such as degradation, cellular penetration, and targeted transport of dsRNA. The range of nanomaterials is diverse, including chitosan, liposomes, inorganic materials, synthetic polymers, and peptides, each with unique properties that enhance RNAi efficacy [11]. Among these, carbon nanoparticles have emerged as a promising carrier for dsRNA delivery, offering advantages such as low toxicity, high biocompatibility, and intrinsic fluorescence, which facilitates precise tracking of delivery [12]. Carbon quantum dots (CQDs) have been extensively used to form nanoparticles with dsRNA via electrostatic interactions, thereby gaining prominence in insect RNAi research. CQDs, along with other nanoparticle types, have proven effective in delivering dsRNA for the management of plant pathogens and pests [13]. Due to their robust endosomal escape capabilities, CQD nanoparticles have been identified as the most efficient carriers for inducing systemic RNAi through oral administration in *Chilo suppressalis* [14]. Furthermore, carbon dots derived from waste candle soot have been employed to deliver dsRNA targeting storage proteins and vitellogenin, resulting in a significant disruption of growth and reproduction in *Spodoptera litura* [15]. Collectively, these studies underscore the distinct advantages of CQDs in terms of biocompatibility, traceability, and delivery efficiency, highlighting their potential for future agricultural applications.

Chitin biology is central to these developmental processes. As a major structural polysaccharide of the insect exoskeleton and midgut peritrophic matrix, chitin is essential for maintaining cuticle integrity, preventing desiccation and pathogen invasion, and shaping the gut environment for digestion and nutrient absorption [16]. Because insects do not store chitin, its cyclic degradation during molting and morphogenesis must be precisely coordinated through the regulated expression of chitinases (Chts). These enzymes hydrolyze *β*-1,4-glycosidic linkages in chitin and are indispensable for apolysis, ecdysis, pupation, and adult emergence [17,18,19]. The evolutionary conservation of chitin metabolic pathways, coupled with their absence in plants and vertebrates, makes chitinase genes not only tractable for molecular physiological studies but also highly selective targets for RNAi-based intervention [20]. Physiological investigations in multiple insects have revealed the essential and stage-specific roles of different Cht family members. In *Myzus persicae*, knockdown of *MpCht3*, *MpCht5*, *MpCht7*, *MpCht10*, and *MpCht11-2* disrupts nymphal molting and causes high mortality [21]. In *Musca domestica*, silencing *MdCht9* significantly reduces chitin content, induces over 50% lethality, and produces wing deformities, underscoring the role of chitin remodeling throughout metamorphosis [22]. In *Sogatella furcifera*, RNAi against several *Cht* genes decreases female fecundity [23]. Comparable effects, including molting defects, growth retardation, and death, are reported in *Leptinotarsa decemlineata* [24], *Phenacoccus solenopsis* [25], and *Bactrocera dorsalis* [26]. Beyond RNAi, chitinase genes have also been exploited to enhance viral biocontrol agents; incorporation of a *Spodoptera exigua Cht* gene into *Hyphantria cunea* nucleopolyhedrovirus markedly increases pathogenicity toward larval hosts [27]. Together, these findings demonstrate the centrality of chitinases in developmental physiology and highlight their potential in novel pest-management strategies.

Despite extensive work in other lepidopterans, the chitinase gene family of *T. absoluta*, including gene repertoire, expression dynamics, and functional roles during molting and metamorphosis, remains unexplored. Such knowledge is crucial not only for designing RNAi-based control tools but also for understanding the physiological mechanisms that underpin the species’ rapid development, high survivorship, and ecological adaptability. Therefore, this study provides the first comprehensive identification and bioinformatic characterization of the chitinase gene family in *T. absoluta*. We examine temporal and tissue-specific expression patterns across key developmental transitions, with a focus on physiological processes related to cuticle turnover, metamorphic remodeling, and digestive system restructuring. To functionally dissect these genes, we employ stage-appropriate RNAi delivery strategies: (1) microinjection into pupae, which is an immobile, rigid stage well suited for precise dsRNA administration, to evaluate effects on metamorphic progression, pupal development, and adult eclosion; and (2) a carbon quantum dot (CQD)-mediated soaking method for feeding larvae, enabling efficient dsRNA uptake to assess consequences for larval survival, cuticle integrity, and molting success. The latter also offers a potential platform for oral RNAi delivery in future applied contexts. We believe that through these integrated molecular and physiological approaches, we elucidate the indispensable roles of multiple chitinase genes in *T. absoluta* development and metamorphosis. Our findings not only advance the basic physiological understanding of chitin metabolism in a globally important invasive species but also provide a mechanistic foundation and technical pathway for designing stage-targeted RNAi strategies. Such strategies may contribute to more sustainable, biologically grounded management options for *T. absoluta*, aligning with current demands for precision pest control that minimizes environmental impact.

## 2. Materials and Methods

### 2.1. Insect Rearing

A laboratory colony of *T*. *absoluta* was initially established from individuals collected in 2023 from Kunming City, Yunnan Province, China. The colony was continuously maintained on tomato plants (*Solanum lycopersicum* cv. Provence) under controlled environmental conditions of 26 ± 1 °C, 60 ± 5% relative humidity, and a photoperiod of 16 h light followed by 8 h darkness, as reported before [28]. These conditions ensured stable development and synchronized life stages for downstream physiological and molecular experiments.

### 2.2. RNA Isolation and cDNA Synthesis

Total RNA was extracted from all developmental stages and tissues using the TaKaRa MiniBEST Universal RNA Extraction Kit (TaKaRa, Dalian, China), following the manufacturer’s protocol. RNA concentration and purity were determined using a NanoDrop 2000 spectrophotometer (Thermo Fisher Scientific, Waltham, MA, USA), and RNA integrity was evaluated via electrophoresis on 1% agarose gels. First-strand cDNA synthesis was performed using 1 µg of total RNA and the One-Step gDNA Removal and cDNA Synthesis SuperMix (TransGen Biotech, Beijing, China). The resulting cDNA samples were stored at −80 °C for subsequent use.

### 2.3. Identification and Phylogenetic Analysis

To identify *chitinase* (*Cht*) family genes in *T. absoluta*, raw RNA-sequencing reads were initially obtained from the National Center for Biotechnology Information (NCBI) Sequence Read Archive (SRA) database (Accession number: SRR13065833). A de novo transcriptome assembly was conducted using the SOAPdenovo-Trans version 1.04 [29]. Subsequently, a local BLAST search (https://blast.ncbi.nlm.nih.gov/Blast.cgi, accessed on 5 November 2025) was performed against all assembled unigenes utilizing TBtools version 2.156. Reference chitinase sequences from other lepidopteran species, including *Bombyx mori* and *Plutella xylostella*, were retrieved from the NCBI database and used as query sequences. This search resulted in the identification of eleven putative *Cht* family genes in *T. absoluta* (Table S1). Candidate open reading frames (ORFs) were PCR-amplified using gene-specific primers (Table S2), cloned into the pTOPO-TA/Blunt vector (Aidlab Biotech, Beijing, China), and transformed into *Escherichia coli DH5α* competent cells (Yeasen Biotech, Shanghai, China). Positive clones were sequenced by CoWin Biotech (Taizhou, China). The ORF sequences were verified with ORF finder (http://www.ncbi.nlm.nih.gov/gorf/gorf.html, accessed on 5 December 2025). Domain structures were predicted by using SMART (https://smart.embl-heidelberg.de/, accessed on 5 December 2025) and the NCBI Conserved Domain Search (https://www.ncbi.nlm.nih.gov/Structure/cdd/wrpsb.cgi, accessed on 5 December 2025).

To examine evolutionary relationships among chitinases, amino acid sequences from Lepidoptera, Coleoptera, Diptera, and Hemiptera were collected from GenBank (Table S3). Multiple protein sequence alignments were generated using ClustalW in MEGA 7, after which a phylogenetic tree was constructed using the neighbor-joining method with 1000 bootstrap replicates to evaluate node confidence [30]. This comparative analysis enabled classification of *T. absoluta Cht* genes into established subfamilies and provided insight into their evolutionary divergence.

### 2.4. Developmental and Tissue-Specific Expression Analysis

Expression profiling was conducted across major developmental stages, including first- through fourth-instar larvae, prepupae, pupae (1–7 days old), and adults (1–5 days old). Six larval tissues, including head, integument, fat body, Malpighian tubules, foregut, and midgut, were dissected from fourth-instar larvae under a stereo microscope (Olympus, Tokyo, Japan). Total RNA extraction and cDNA synthesis followed the procedures outlined in Section 2.2. Quantitative real-time PCR (qPCR) was performed on a CFX-96 real-time detection system (Bio-Rad, Hercules, CA, USA). Each reaction (20 µL) contained 10 µL of TransStart^®^ Top Green qPCR SuperMix (TransGen Biotech, Beijing, China), 1 µL of cDNA template, 1 µL of each of forward and reverse primers (Table S2), and 7 µL of nuclease-free water. Thermal cycling conditions included an initial denaturation at 95 °C for 30 s, followed by 41 cycles of 95 °C for 5 s and 55.9 °C for 30 s. Melting curve analysis (60–95 °C) confirmed primer specificity. Relative expression levels were calculated using the 2^−ΔΔCt^ method [31], with *elongation factor 1-alpha* (*TaEF1α*; GenBank accession number: MZ054826) serving as the internal reference [32].

### 2.5. Functional Analysis of Chitinase Genes via RNAi

To investigate the functional roles of chitinase family members during *T. absoluta* development, dsRNA targeting specific *TaCht* genes was synthesized using the TranscriptAid T7 High Yield Transcription Kit (Thermo Fisher Scientific, Wilmington, DE, USA). The ds*GFP* served as the negative control. For pupal-stage RNAi assays, 2-day-old pupae were immobilized and microinjected with 500 ng of dsRNA into the dorsal vessel of the second dorsal segment using a Nanoliter 2010 microinjector (World Precision Instruments, Sarasota, FL, USA). Each treatment contained 30 pupae, with four biological replicates. Injected pupae were transferred to sterile soil moistened with sterile water and maintained under standard conditions until adult emergence or mortality assessment.

RNAi at the larval stage employed a CQD-mediated soaking method to enhance dsRNA uptake, and the CQDs were prepared as previously described [33]. Uniformly developed third-instar larvae were starved for 4 h before treatment. Larvae were then immersed in a dsRNA-CQD mixture prepared at a dsRNA: CQD ratio of 2:1, yielding a final dsRNA concentration of 1000 ng/µL in the soaking solution. After 10 min of incubation, the mixture was removed with a micropipette, and larvae were transferred onto fresh tomato leaves for rearing under the same conditions described in Section 2.1. To assess knockdown efficiency, 20 larvae were randomly selected at 72 h post-treatment for qPCR analysis. The remaining larvae were monitored every 24 h for 8 days to document survival rates and morphological abnormalities. Morphological observations were conducted using a Keyence digital microscope (Keyence, Osaka, Japan), allowing detailed assessment of cuticle defects, molting failure, and developmental arrest.

### 2.6. Chitinase Activity Assay

To evaluate how *TaCht* silencing affects endogenous chitinase activity in *T. absoluta* pupae, a quantitative enzymatic assay was conducted using the Chitinase Assay Kit (Solarbio, Beijing, China). Pupal samples were collected 24 h after dsRNA injection. Approximately 0.1 g of pupal tissue was homogenized in 1 mL of cold extraction buffer, followed by centrifugation at 10,000× *g* for 20 min at 4 °C. The supernatant was collected for chitinase activity measurement. A standard curve was prepared using serial dilutions of *N*-acetyl-*D*-glucosamine. Chitinase activity was calculated based on the concentration of *N*-acetylglucosamine produced in the reaction and expressed as units per gram (U/g) of tissue. One unit (U) of enzyme activity was defined as the amount of enzyme capable of generating 1 µg of *N*-acetylglucosamine per hour at 37 °C. The calculation used was: Chitinase activity (U/g) = 2.5 × C/W, where C is the *N*-acetylglucosamine concentration (µg/mL) determined from the standard curve and W is the tissue mass (g). Each treatment included three biological replicates, each with three technical repeats.

### 2.7. Frozen Sectioning and H&E Staining

To investigate structural alterations in the larval cuticle following chitinase gene knockdown, frozen sectioning followed by hematoxylin-eosin (H&E) staining was performed. Larvae were collected 24 h after dsRNA treatment and fixed overnight in 4% paraformaldehyde at room temperature. After dehydration, samples were embedded in frozen embedding medium and placed in a cryostat microtome for 30 min before sectioning. Tissue sections (10 µm) were mounted onto adhesive slides. Sections were stained with hematoxylin for 60 s, rinsed, and differentiated in 0.1% hydrochloric acid-ethanol until nuclei appeared purple. Bluing was performed in PBS/warm water/1% ammonia for 50 s, followed by eosin staining for 60 s. The slides were dehydrated in 95% ethanol twice for 5 min each, cleared in xylene twice for 5 min each, and examined using a LSM 900 confocal laser-scanning microscope (Zeiss, Oberkochen, Germany).

### 2.8. Statistical Analysis

Survival curves were analyzed using the Kaplan–Meier method, and differences between ds*TaCht*-treated and control groups were tested using the Log-rank test. For pairwise comparisons between RNAi-treated and control samples, Student’s *t*-test was used (* *p* < 0.05; ** *p* < 0.01). All statistical analyses were performed in SPSS v26.0, and figures were generated using GraphPad Prism v8.3.

## 3. Results

### 3.1. Molecular Cloning and Sequence Analysis of Chitinase Genes

**Figure 1:**
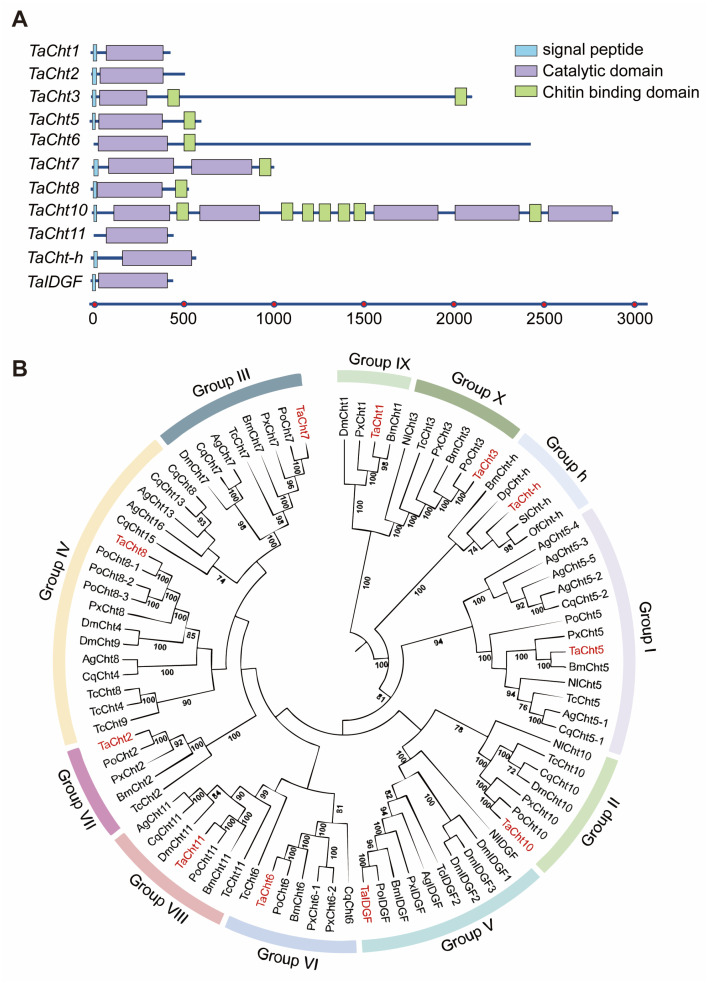
Domain and phylogenetic analysis of chitinase proteins in *T. absoluta*. (**A**) Domain architectures of eleven chitinases in *T. absoluta*. Domain structures were predicted using SMART and the NCBI Conserved Domain Search based on deduced amino acid sequences. Signal peptides are shown in blue, catalytic domains in purple, and chitin-binding domains in green. (**B**) Phylogenetic analysis and group classification of chitinases (Chts) from *Anopheles gambiae* (Ag), *Bombyx mori* (Bm), *Culex quinquefasciatus* (Cq), *Danaus plexippus* (Dp), *Drosophila melanogaster* (Dm), *Nilaparvata lugens* (Nl), *Ostrinia furnacalis* (Of), *Phthorimaea operculella* (Po), *Plutella xylostella* (Px), *Spodoptera litura* (Sl), *Tribolium castaneum* (Tc), and *Tuta absoluta* (Ta). *T. absoluta* chitinases are indicated by red dots. Phylogenetic tree was constructed by MEGA 7 using the neighbor-joining method. Bootstrap analyses with 1000 replications were conducted, and only node support values of >70% are shown. The GenBank accession numbers for these chitinases are listed in Table S3.

From the *T. absoluta* transcriptome database, 11 full-length cDNA sequences belonging to the chitinase gene family were successfully cloned and identified: *TaCht1*, *TaCht2*, *TaCht3*, *TaCht5*, *TaCht6*, *TaCht7*, *TaCht8*, *TaCht10*, *TaCht11*, *TaCht-h*, and *TaIDGF*. The ORFs of these genes ranged from 1182 to 8667 bp. Domain prediction analyses revealed that all TaCht proteins contain conserved glycosyl hydrolase family 18 (GH18) catalytic domains (Figure 1A). Several chitinases contained multiple GH18 copies, with TaCht7 harboring two and TaCht10 possessing five such domains, whereas the remaining proteins each contained one. Signal peptides were predicted at the N-termini of all chitinases except TaCht6 and TaCht11, suggesting that most TaChts may be secreted enzymes. Chitin-binding domains (CBDs) were present in TaCht3, TaCht5, TaCht7, TaCht8, and TaCht10, with TaCht3 containing two CBDs and TaCht10 containing seven, indicating potentially strong associations with chitinous substrates. Phylogenetic analysis clustered the 11 TaChts into distinct, well-supported groups corresponding to classical insect chitinase subfamilies (Figure 1B). TaCht5 belonged to Group I, TaCht10 to Group II, TaCht7 to Group III, TaCht8 to Group IV, TaIDGF to Group V, TaCht6 to Group VI, TaCht2 to Group VII, TaCht11 to Group VIII, TaCht1 to Group IX, and TaCht3 to Group X. TaCht-h, a lineage-specific chitinase found only in Lepidoptera, was placed within the lepidopteran-specific h-group.

### 3.2. Developmental and Tissue-Specific Expression Patterns

**Figure 2:**
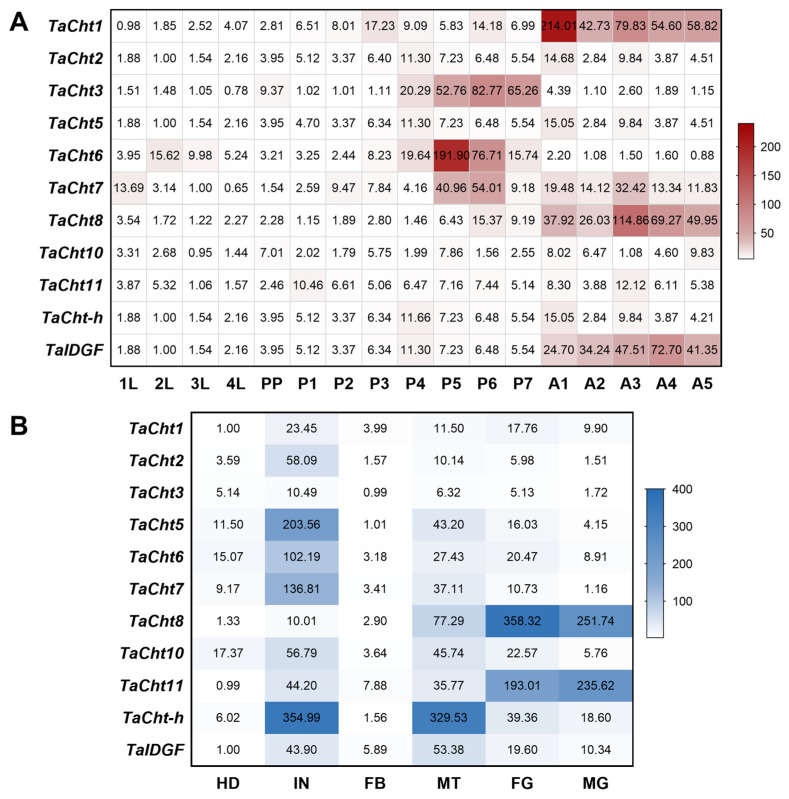
Spatiotemporal expression profiles of chitinase genes in *T. absoluta*. (**A**) Temporal expression patterns of *TaCht* genes across developmental stages. Whole bodies from seventeen stages were used for qPCR analysis. L1–L4: first- to fourth-instar larvae; PP: prepupae; P1–P7: pupae 1 to 7 days old; AD1–AD5: adults 1 to 5 days old. (**B**) Tissue expression profiles of *TaChts* in fourth-instar larvae. cDNA was prepared from the head (HD), integument (IN), fat body (FB), Malpighian tubules (MT), foregut (FG), and midgut (MG). Table S4 presents the expression values of each gene across various developmental stages and tissues.

Expression profiling across developmental stages showed that *TaCht1*, *TaCht2*, *TaCht5*, *TaCht8*, *TaCht10*, *TaCht11*, *TaCht-h*, and *TaIDGF* were highly expressed during the adult stage (Figure 2A). Among these, *TaCht1*, *TaCht2*, *TaCht5*, and *TaCht-h* exhibited peak expression on the first day post-eclosion, while *TaCht8* and *TaCht11* reached maximal expression on day three. *TaCht3*, *TaCht6*, and *TaCht7* displayed predominantly pupal-biased expression, with *TaCht3* showing a gradual increase from days 4 to 7, and *TaCht6* and *TaCht7* peaking on days 5 and 6, respectively.

Tissue-specific profiles indicated that most genes, including *TaCht1*, *TaCht2*, *TaCht3*, *TaCht5*, *TaCht6*, *TaCht7*, *TaCht10*, *TaCht-h*, and *TaIDGF*, were preferentially expressed in the integument, supporting their involvement in cuticle turnover during molting (Figure 2B). In contrast, *TaCht8* and *TaCht11* showed highest expression in the gut, suggesting functional roles in peritrophic matrix remodeling or digestive processes. *TaCht-h* and *TaIDGF* also exhibited elevated expression in the Malpighian tubules, while *TaCht3* displayed notably high expression in the head, potentially indicating additional roles in mouthpart remodeling or neural-associated structures.

### 3.3. Silencing Efficiency and Mortality Induced by RNAi of Chitinase Genes

**Figure 3:**
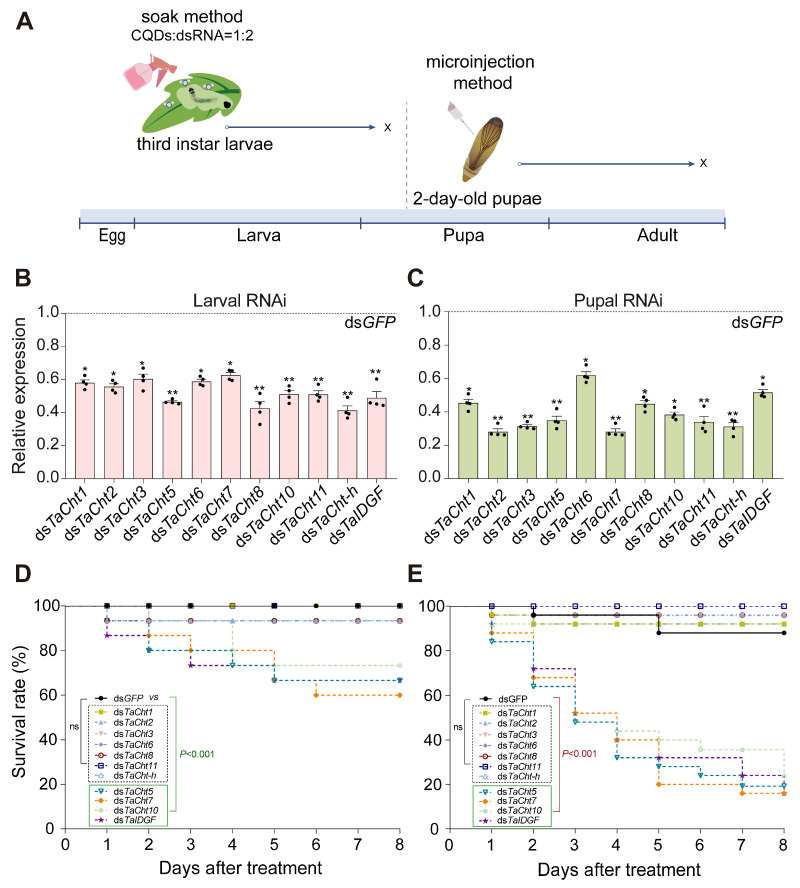
Effect of *TaCht* gene knockdown on survival in *T. absoluta*. (**A**) Schematic of the RNAi strategy: third-instar larvae ingest leaves pre-soaked with a nanomaterial/dsRNA complex for *TaCht* silencing, whereas 2-day-old pupae receive direct microinjection of dsRNA. (**B**) Relative expression of *TaChts* in third-instar larvae 3 days after ds*TaChts* or ds*GFP* soaking. (**C**) Relative expression of *TaChts* in 2-day-old pupae 3 days after ds*TaChts* or ds*GFP* microinjection. (**D**) Effect of *TaCht* silencing on 8-day survival of third-instar larvae after dsRNA soaking. (**E**) Effect of *TaCht* silencing on 8-day survival of 2-day-old pupae after dsRNA microinjection. Significance was determined using Student’s *t*-test (** *p* < 0.01, * *p* < 0.05, ns: no significance). Mean survival time was calculated using the Kaplan–Meier method.

RNAi-mediated knockdown of individual *TaCht* genes was performed to investigate their essential biological roles. During the larval stage, third-instar larvae were exposed to CQD-assisted dsRNA soaking (Figure 3A). The qPCR analyses at 72 h post-treatment verified that transcript levels of all 11 *TaCht* genes were significantly reduced (Figure 3B). Survival analysis revealed that silencing *TaCht5*, *TaCht7*, *TaCht10*, and *TaIDGF* resulted in substantial larval mortality, namely 67%, 60%, 73%, and 67%, respectively, while knockdown of the remaining seven chitinase genes did not significantly affect larval survival (Figure 3D).

In the pupal stage, microinjection of dsRNA into 2-day-old pupae successfully induced strong transcript suppression of all target genes at 72 h (Figure 3C). Consistent with larval results, silencing of *TaCht5*, *TaCht7*, *TaCht10*, and *TaIDGF* significantly increased pupal mortality, whereas silencing the other *TaCht* genes produced no significant effects (Figure 3E). These findings demonstrate that these four genes play indispensable roles during both larval and pupal development, while other chitinases may have redundant or stage-limited functions.

### 3.4. Effects of Silencing TaCht5, TaCht7, TaCht10, and TaIDGF on Larval–Pupal Ecdysis

**Figure 4:**
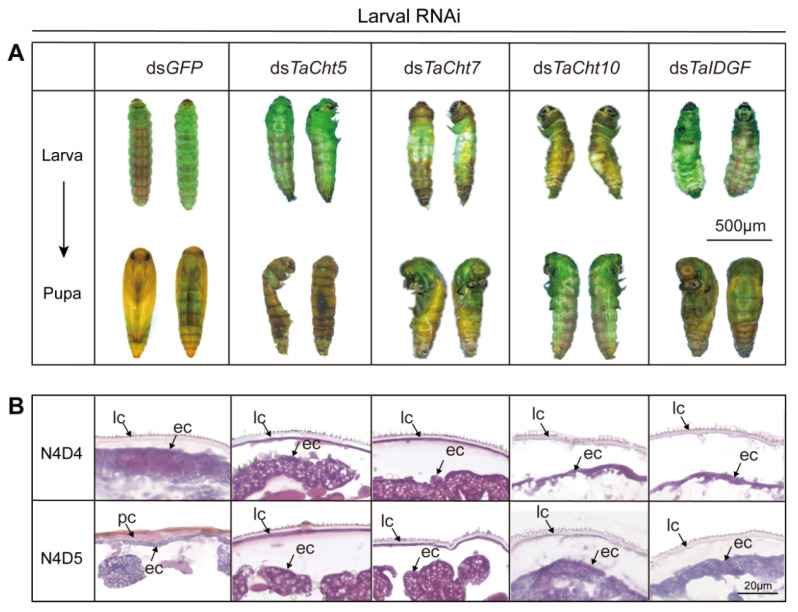
Effects of silencing *TaCht5*, *TaCht7*, *TaCht10*, and *TaIDGF* on larval–pupal development in *T. absoluta*. (**A**) Defective larval phenotypes following knockdown of *TaCht5*, *TaCht7*, *TaCht10*, and *TaIDGF*. (**B**) Hematoxylin and eosin staining of abdominal cuticle sections after injection with dsRNA targeting *TaCht5*, *TaCht7*, *TaCht10*, *TaIDGF* or ds*GFP*. ec: epidermal cells; lc: larval cuticle; pc: pupal cuticle.

To clarify how these four genes contribute to larval–pupal transition, third-instar larvae were treated with dsRNA-CQD complexes. In the ds*GFP* control group, larvae successfully completed larval–pupal ecdysis (Figure 4A). In contrast, ds*TaCht5*-treated larvae displayed severe shrinkage, cuticular melanization, and ultimately death without successful molting. Silencing *TaCht7*, *TaCht10*, or *TaIDGF* produced similar phenotypes, including pronotal cracking, failed separation of the abdominal integument, and incomplete apolysis that prevented pupation (Figure 4A).

The observed mortality rates, namely 67% for ds*TaCht5*, 60% for ds*TaCht7*, 73% for ds*TaCht10*, and 67% for ds*TaIDGF*, corroborated the phenotypic severity of molting defects. To further examine cuticular changes, H&E staining revealed that control larvae exhibited clear separation between the new and old cuticle layers prior to ecdysis. In contrast, dsRNA-treated larvae showed detachment of epidermal cells from the cuticle, irregular chitin organization, and failed cuticle splitting (Figure 4B), explaining the lethal molting failure induced by gene silencing.

### 3.5. Effects of Silencing TaCht5, TaCht7, TaCht10, and TaIDGF on Pupal–Adult Transition

**Figure 5:**
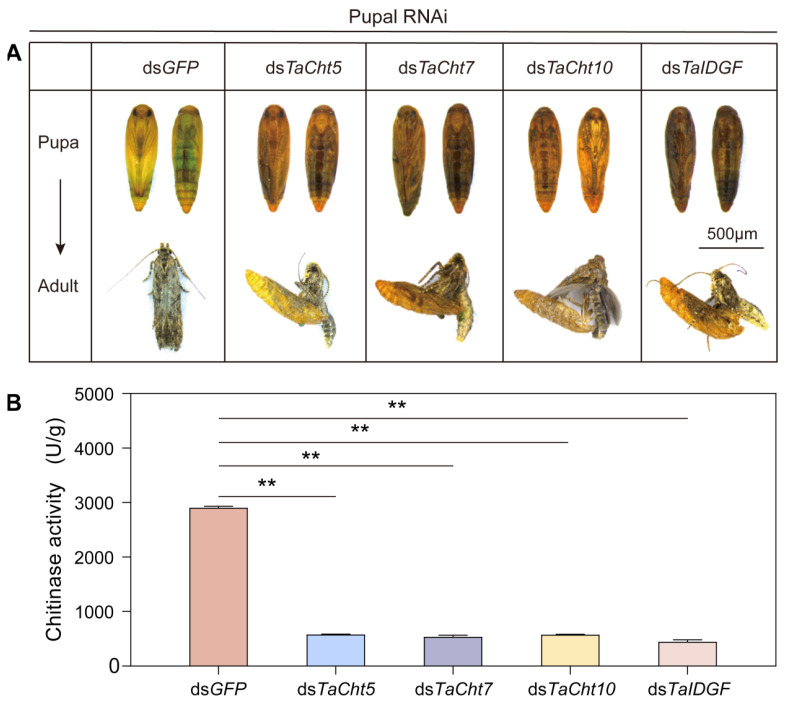
Effects of silencing *TaCht5*, *TaCht7*, *TaCht10*, and *TaIDGF* on pupal–adult development in *T. absoluta*. (**A**) Defective pupal phenotypes following knockdown of *TaCht5*, *TaCht7*, *TaCht10*, and *TaIDGF*. (**B**) Changes in chitinase activity after injection of dsRNA targeting *TaCht5*, *TaCht7*, *TaCht10*, *TaIDGF* or ds*GFP*. Significance was determined using Student’s *t*-test (** *p* < 0.01).

To evaluate the functions of these genes in pupal metamorphosis, dsRNAs were microinjected into 2-day-old pupae and developmental outcomes were observed. After ds*TaCht5* injection, 67% of pupae underwent cuticular melanization and died before eclosion, and an additional 13% initiated eclosion but could not shed the pupal case, resulting in death. Silencing *TaCht7* caused 59% melanized, shrunken pupae and 23% adults that reached eclosion but failed to detach from their pupal cases. In ds*TaCht10*-treated pupae, 55% exhibited shrinkage and melanization, and 15% died during incomplete eclosion. Silencing *TaIDGF* caused 61% early pupal mortality due to melanization and 25% mortality during incomplete adult emergence. The total mortality rates were 81% (*TaCht5*), 84% (*TaCht7*), 76% (*TaCht10*), and 84% (*TaIDGF*), while all ds*GFP*-injected pupae successfully emerged as normal adults (Figure 5A). Chitinase activity assays showed that 72 h after dsRNA injection, enzymatic activity decreased by 82% in ds*TaCht5*-, 80% in ds*TaCht7*-, 85% in ds*TaCht10*-, and 80% in ds*TaIDGF*-treated pupae compared to controls (Figure 5B).

## 4. Discussion

Chitinases are essential enzymes involved in chitin degradation, playing indispensable roles in insect growth, molting, and metamorphosis. Consequently, the identification and characterization of insect chitinases are crucial for uncovering developmental mechanisms while simultaneously guiding the design of innovative, physiology-based pest control tools. In this study, eleven chitinase genes were identified from the transcriptome database of *T. absoluta*, representing a moderate gene family size relative to other insects. Comparable chitinase gene numbers have been reported in *Acyrthosiphon pisum* and *B. mori*, both with nine genes [34,35], *Spodoptera frugiperda* with eleven [36], and *P. xylostella* with thirteen [37]. In contrast, species such as *L. decemlineata*, *S. furcifera*, and *Aedes albopictus* contain 12, 19, and 20 chitinase genes, respectively [24,38,39]. These interspecific differences highlight lineage-specific expansions potentially associated with distinct ecological niches, cuticle chemistries, or metamorphic strategies.

Phylogenetic analysis confirmed that the identified *T. absoluta* chitinases follow the conventional classification for Lepidoptera [37], placing all TaChts into groups I-X and h. The conservation of GH18 catalytic domains across all identified genes, accompanied by diverse domain architectures [40], suggests functional partitioning of chitinolytic processes across tissues and developmental stages. This structural diversification aligns with the increasing appreciation within insect physiology that chitinase paralogs evolve distinct biochemical properties to meet the variable mechanical and physiological demands of molting, gut homeostasis, immune defense, and cuticle remodeling.

The expression patterns of chitinase genes across developmental stages and tissues in insects typically reveal strong specialization. Previous investigations, for example, in *S. frugiperda* and *Diaphorina citri*, have demonstrated integument-specific expression of several *Cht* paralogs (*SfCht2*, *SfCht3*, *SfCht10*), midgut-enriched expression of *Cht8* subfamily members, and stage-specific peaks aligned with larval–larval or larval–pupal molts [36,41]. The *T. absoluta* expression profiles observed here reinforce these patterns, offering new insights into cuticle physiology within this highly invasive pest. Elevated expression of *TaCht1*, *TaCht2*, *TaCht5*, *TaCht7*, *TaCht10*, *TaCht-h*, and *TaIDGF* during molting, coupled with their enrichment in the integument, strongly suggests involvement in cuticular turnover. Conversely, gut-specific expression of *TaCht8* and *TaCht11* indicates potential roles in peritrophic matrix remodeling or digestive physiology, opening avenues for further functional dissection of midgut-specific chitin synthases and hydrolases.

A major innovation of this study lies in the development and application of stage-tailored RNAi delivery systems, addressing a long-standing limitation in insect functional genomics. Physiological barriers such as gut nucleases, cuticle hardening, and feeding cessation have historically constrained the efficacy of RNAi in holometabolous insects [7]. By deploying nanocarrier CQDs during the larval stage, which is when feeding and gut permeability favor dsRNA uptake, and microinjection during the nonfeeding pupal stage, which is when direct hemocoelic access is required, this work demonstrates a robust methodology capable of overcoming these barriers [8]. The dual approach not only enhances gene-silencing efficiency but also provides a blueprint for integrating nanoparticle-assisted delivery with conventional techniques across metamorphosis, marking a significant achievement in insect physiological experimentation. Notably, CQDs can be synthesized from cost-effective carbon precursors and readily conjugated with dsRNA, suggesting the technical feasibility of large-scale production [42,43]. Within this context, CQD-dsRNA complexes hold potential for adaptation to foliar applications, such as direct spraying onto plant surfaces, thereby exposing feeding larvae to dsRNA in a manner similar to other nano-enabled RNAi strategies documented for crop protection [44]. However, these applications are currently speculative and necessitate comprehensive validation.

The functional assays focusing on the larval–pupal and pupal–adult transitions revealed striking phenotypic effects associated with four genes: *TaCht5*, *TaCht10*, *TaCht7*, and *TaIDGF*. The absence of phenotypes following the silencing of *TaCht1*, *TaCht2*, *TaCht3*, *TaCht6*, *TaCht8*, and *TaCht11* suggests functional redundancy or roles limited to physiological processes not critical for molting survival. These findings parallel studies in *S. furcifera* where several *Cht* paralogs showed no lethal phenotypes upon RNAi despite detectable expression [38], emphasizing the need for broader integrative analyses of gene networks, compensatory metabolic pathways, and hormonal regulation. In contrast, the lethal molting defects observed after silencing *TaCht5*, *TaCht7*, *TaCht10*, and *TaIDGF* underscore the essential functions of these chitinases in cuticular degradation. Massive evidence across insect orders supports the fundamental involvement of *Cht5*, *Cht7*, and *Cht10* families in ecdysis-driven remodeling [45]. Orthologous phenotypes include failed nymph–adult transition in *S. furcifera* [38], abnormal pupal eclosion in *P. xylostella* [37], wing-curling defects in *C*. *suppressalis* [46], compromised cuticle integrity and flight morphology in *D. melanogaster* [47], and larval mortality in *Agrotis ipsilon* due to suppressed chitin degradation [48]. The parallels in *T. absoluta*, which included melanization, integument shrinkage, arrested ecdysis, and fatal entrapment within exuviae, confirm that chitinase-mediated cuticle turnover is physiologically conserved but anatomically tuned to species-specific cuticle architectures. Microscopic H&E analysis provided direct evidence linking gene silencing to cellular disruptions, revealing failures in epidermal-cuticular detachment, a key prerequisite for successful apolysis. Coupled with significant reductions in enzymatic activity following dsRNA treatment, these findings create a mechanistic bridge connecting gene expression, enzyme function, and whole-organism outcomes. Such multi-level validation represents a major strength of this study, emphasizing integrative approaches bridging molecular, cellular, and organismal physiology.

Next to the fundamental insights, we believe that the applied implications of these findings are substantial. Chitinases central to metamorphosis, particularly *TaCht5*, *TaCht7*, *TaCht10*, and *TaIDGF*, represent high-value RNAi targets for next-generation biopesticides. The demonstrated efficiency of CQD-assisted RNAi uptake in larvae offers a feasible route for field-adapted formulations, and the species specificity of chitinase sequences minimizes risks to beneficial arthropods. Moreover, because chitin metabolism is highly conserved yet absent in plants and vertebrates, targeting these genes avoids hazards associated with off-target effects typical of chemical insecticides. However, several limitations and knowledge gaps remain. First, while this study documents lethal phenotypes, it does not quantify potential compensatory upregulation of other chitinases or related hydrolases following RNAi, a process increasingly recognized in RNAi-mediated gene networks. Second, upstream endocrine regulation, particularly 20E and juvenile hormone signaling, was not explored, limiting interpretation of how *TaCht* expression integrates into larger metamorphic circuits. Third, the efficiency of CQD-dsRNA complexes under natural environmental conditions (UV degradation, microbial activity, plant leaf surface chemistry) remains untested. Finally, although microinjection offers strong mechanistic insight, it is impractical for field application, necessitating the development of environmentally stable larval-delivery formulations. Based on this, we suggest that future directions should include: (1) integrative hormonal and transcriptomic profiling during silencing to map chitinase regulation within the broader endocrine landscape; (2) CRISPR-based knockout studies to validate essential functions without RNAi variability; (3) structural enzymology to identify catalytic residues and potential inhibitors; (4) evaluation of nanoparticle-based oral RNAi in greenhouse and field conditions; and (5) exploration of synergistic pest control strategies, combining RNAi with fungal entomopathogens, botanical compounds, or NPV-based approaches [22]. Collectively, our study provides new mechanistic insights into the physiology of insect molting and metamorphosis, advances methodological innovation for functional genomics in holometabolous insects, and establishes chitinase pathways as promising and practical targets for next-generation pest management strategies.

## Data Availability

The original contributions presented in this study are included in the article/Supplementary Materials. Further inquiries can be directed to the corresponding authors.

## Supplementary Materials

The following supporting information can be downloaded at: https://www.mdpi.com/article/10.3390/insects17010114/s1, Table S1: Putative nucleotide sequences of eleven chitinase family genes in *Tuta absoluta*. Table S2: Primers used in this study. Table S3: Scientific names of chitinase sequences from different insect species used in constructing the phylogenetic tree. Table S4: Developmental and tissue-specific expression profiles of eleven *TaCht* genes in *Tuta absoluta*.
