# Supplementary material for: Identification and Functional Characterization of Chitinase Genes During Larva–Pupa–Adult Transitions in Tuta absoluta
Source: Insects. 2026 Jan 20;17(1):114. doi: 10.3390/insects17010114 (PMC12841776; doi:10.3390/insects17010114)
Supplement: Supplementary file 1 [file insects-17-00114-s001.zip › Table S1.pdf]

Table S1. Putative nucleotide sequences of eleven chitinase family genes in *Tuta absoluta*.

> TaCht1 [organism=*Tuta absoluta*] chitinase 1 mRNA
ATGCAATGTCTGAAGACGTTACTTCAAGTTATATTTCTTTTTACTGTTTGTTTGGCGACGCTATCGCC
TCCGTCCGATAAGAAGGGGCAGAAAGGAGTAAAACCTCAGGAAGGGCCTAGGAAAAACAATGTTT
TGGAGAGGAAACTAGTAGTAGAGACACCGCTAGTCAAAGATATCCTGAAGTACCACGCTACGTACC
ACCAGGATGTGGCTTCTCGGAATTTCAACAACAAAGTTCTTGGTTACGTTACGCCGTGGAACAGCA
AGGGCTATGATGTTGCAAAAACCTGGGCTCCAAAGTTCAACTACATTTCACCAGTGTGGCTGCAGA
TCAAGAGGCAGTCCCCGAACATCTACATCACATCCGGTCTCCATGACGTCGACCATGCCTGGATGA
AGAGTGTGCGGCAAAAGGGAGAGAATAATAATATTAAAATAATGCCCAGAATGATATTTGAAAAC
TGGCAGTTAACAGATCTGAAAGTGTTCTTCATGGAGCCAACGTCCCACTCAGAACAGAAGGCTCTC
ATTGAGGAGGTGAAGAAAATCTGTAAGCAGTGGAAGTTTGACGGCATTGTTCTTGAAATGCTCTCT
CAGATTGGCAAGTATGCTGATCGATCTGTCAAGTTCATTCAGCAGCTTGGTATTGAAATGAATTTAG
ATGATCTCAGTTTGATCCTAGTCTACCCACCTTTCCGGGGGTATCCCACAGATGAGTTTTTCATACA
AGCATTTAATGATATTTACCCATATGTAGAAGCTGTGTCTGTGATGACTTATGACTTCTCTAATGCCC
AGAAACCAGGTCCAAATGCTCCTATTTCCTGGATGAGGCTTTGTGTAGAAAAGCTCTTAGATGGAG
ATGACAGTCAAAAGCGTTCAAAAATCCTTTTAGGAATGAATTTCTATGGTAATTCCTACACGGCCAA
TGGTGGCGGTCCCATTGTAGCGACCGAGTATATTGAGTTGTTGAAATATGCCAAGAATAACGCGGC
TGTAACGTATAATAATAATACAGCTGAGAATTATTTAGAAGTCAAGACTTCGCAAGGAACCAAGAA
AATATTCTACCCCACTCTGTTTTCTATACAGAAAAGACTCGATTTGGCGCGCGAGTATGGAACCGGA
ATCGCTATATGGGAAATTGGTCAAGGCCTCGACTATTTTTATGATTTATTTTGA

> TaCht2 [organism=*Tuta absoluta*] chitinase 2 mRNA
ATGGAGAAAAATATAAGTTTGTGTTGTTTGTTCGTTGTTTTTATAGTTTTGACCGTGGCTTCTGTTAT
GGATGGACAAACCTTGGGTGGACCTATGCACGGCAAGGTAGTGGTCTGCTACGTAGCGACATGGGC
GGTCTACCGGCCGGAGCCCGGCAGCTTCACGCTGGAAGACATCGATCCCTCACTCTGCACCCATTTG
GTCTACTCCTTCGCCGGGCTGGACGAGGCTACCGGTGGCATCAAGAGCCTGGATGCGTGGCAGGAC
CTGGAGAAAGACTACGGCAAGGCTGGCTTCAAGCGGATAGTGAACCTGAAGCATCGCTATCCTCAC
CTCAAGGTCACCATCGCCATCGGGGGCTGGAATGAGGGGTCCTACAAGTACTCCAAGATGGCCGAG
ACTCCTGAGACCAGGGCCAAGTTCATCAAGAGTGTGCTGCTGTTCTTGGAGACGTACAAGTTCGAT
GGCCTAGACCTCGACTGGGAGTACCCAGCCAGACGTGACGGCAGACCGATTGATAAAGCCAACTAC
GTTTCCCTCGTGAAGGAGCTGAACGAAGCGTTCGAGCCGCACGGCTACATCCTGACGGCGGCCCTG
GGCGCCGGCAAGGAGACCATGGAGGCGGCGTACGAGCTGACCAAGCTGAGCCGCTACCTGACCCT
CATCCACATGATGTGCTACGACTACCACGGCACCTGGGACGGGGTGGTTGGTGCCAACGCGCCGCT
AACCAGTGCTGATCAAAACGACGTTCTCACTGTGGAGTACACAATCAAGTACATGTTGGCGCACGG
CGTGAGTCCTTACAAGCTGGTGCTTGGGCTTCCGATGTATGGGAGGAACTTCATCCTCCAGGATCCG
GAGACCAGGGAGGTGGAGTTCGGCAGGACGGCCGCCAAGGCGCAAGGGTTCAAAGGGCCGTTCAC
TAAGGAGGCAGGGTTTATGGGGTATAATGAAATCTGCATGGAGTTAGTAAACAAATCCTCCAATTG
GACGCGGCACTGGCACGCCCCCTCTCAGACACCATACTTGAGGGACGGCGATCGCGTCATGTCATA
TGACAACTCCAGATCCATGGCCGCTAAAGTCAAAATGGCGGTGGACTACGGTCTCGGCGGTCTGAT
GGTGTGGAGCATAGACACGGACGACTTCAAGGGCGCGTGCGGACAAGAAGCAGACGCGTACGTCG
ACTTCGTGGCGCGGTACCACAAGATGGCCGACGATCCCGTGCTTTATGAGGCGCTGAAGACGCTGC
AGTTGCCTGATGCGACACGACTCAGCAAACGCACGTTCTACTCGCAATCAAACGGCAAACTACATT
TACGACTCCCGGAACCCGAGTTCAGCAATTACAACCTGATGCGAACCATCAACGACGCAGCCCATT
TAGCGCTAGAAGAGAAACGTATCACCGACGAGATGGAGAACATCGTGCGGACCAATGAGATCGAC
GATAAGCCGCCGAATGGCGCGCGCGGATTGGTCGGTTCAGTCGTCATGCTGTTGTGTGCGTTGGTTT
GCTTATTTTAG

> TaCht3 [organism=*Tuta absoluta*] chitinase 3 mRNA
ATGTGGCTTTGGTTGTTATCCGTCGTTTCCGCGGCGGTATTTTCGGAGGTGAATTCCGCGGGCGCCG
GCGCCGCGCGACTGGTCTGCTACTCCGACGGTTCACGCACGTCAGGATACGATGAGTGCACGCACC
TGGTGTATTCCGGAGACGCGAGAGGAGACCAGCTCGATGTGCTCCTGAAGGAATACAAGAAGAAC
AATCAGAGGCTGAAGGTTTTGCTGAGGGTTGGAGAGGTTGATAAGGACCTCCGCAGCCTCCTGAAG
TCTAAGCACGTCCAGGGTCTGGAGATCCACGATGCCCACAAGTCTCTCAACAGGACCAAGGTGTTG
GAGACGGTGGAAGCAGCTCGTGCTGCCATCAGCTCTTCTGGTGGTGGTCCCCTATTCCTGGCGCTCC
CCGCGCACCCGGAGCTGCTGGCCAAATACTACGACCTTCGAGCCCTGATGAAGAAGGTGGATCTCA
TGATGGTGCAGACCCACGCCCTGGGACACGTGAAGAGGATGACCTACCACCCCAGCAGGCTGTCTG
GAGTGTGGGACATGATGAATACTGACTCCGTGGTAGACTTGGTTATAGGCCTGGGCGTCCCAGCTTC
CAAGATCGTCATCAGCCTTCCAGCTACAGCTCACCGGTTCCACCTTCTCAACGAGACCCTCAGTACC
CCCGGCAGCCCCACCACGGAGGATGACCCAAAGGAGATCGACCAGGCTGAACTCTGCAGGCTGCTG
CAGAAGGGACGCTGGACCCTCGAGAGGGACCAGGATCTGTCAGCGCCTTATGCTTTCAAGGACAAA
ACCTGGATGTCATTTGAGGACTCCTCCTCAGTCGACGTGAAGGGGAAATACGCGCGAGTCCGAGGG
TTGGCTGGTCTTGCTCTTCACAAAGCTGATGAAGACGTGGAGACGCCATGTGGACCCACTCTCAAG
ACCTCGCTGGCTAAAGTGCTGAATCAGCAGAGTCGGGCTCCCAGGGCTGCGGTTCTAAGATCTCTG
GAACACGAGATCCTGTCAGCCCCTCTTCATGCACGAGCTCTCGATGCTTTACAAGTGTCTCCTTACC
GCATCACGCAGATCGTCGACTCAGATGGAGTCATCCACTCCATCAGAGAGGTGTTCGAATTCGACT
GTCCAGCTGGTCTAGCATTCGACTCACGCTACGAAGTGTGCGTGTGGCCAGGAAGTCTACCTGACTC

TCAAGCCTGCCCAGGGTCCTCTGAAATTGCTCCTGTGCCACGAACCAGATTCATCTGTCCTGAACAT
GAGGGTTACTATGCCGACCCCGAAAATTGCCGTTGGTTCTTCGCCTGCCTTGACCACGGGAAGGCAC
CCCTTACAGCCTACGAGTTCCGTTGTCCATTCGGTCTTGGATTCGACGCTGAGAAGTTGAAATGCGA
CTGGCCTTGGTTGGTGCCAGCCTGTGGCAATATTGCCCGATACGAGGCTGAGGCATTCGGATATGG
GGGAGCTGCTTTTAGCGGTGCAACTGGTTTCCAAGGACAGACGGCAGATGCAATCAATATAGCCGC
TCACCAGAGTCTTATTTCTGGAGCCTCCCTAGACAACCACGTCGGAATCCAAAATGGCTTCCTTTCC
CAAGCAGACGCCCTGGATTCCAACTTCATCGCTTCTCAGGATCTTCAAGGTCATGGATTTCAAATTG
GTCATGGTAATGCTGTAACTAGTGGTAATGGTCATGTAGGATTGGTCCAACCGACAAAATACTCAA
GTGGGTCTATCATCCTTGATGACTACAGGCTGCCAATTAAGGGTGTCGGACACATAGGTGTACAAG
ACGGAGGAATTGGTCATCAAAACGGAGGACAAAGTTATCAAAATGCTGGACAAGGTCTTCAAAAC
GCCTACAGTGGATACGCATCTTTCGGAACTAATGGAAAGAAAGTTCAGTACCAAAGTTCAGATTAT
TCTGATAACTCTGGTGCCTATGTTCACGATCCTACAGGAGACTACGCTGAACCCTACAGACATGTCG
ATTCTCCAGTTGTTCCTTACATACACACCGACTCCAAAACCATTGAATATAATGACTACGGAAGGTA
TTCCGGTACCCATGATTACAACGGTCAATATGCTCATGATAATTCTGGTTCTTATAAAGAAAATAAT
GGATATTCAGGGTCATATAATGCTGACTACTCTGGATCTTATTCTAATGATAATTCCGGAAAATACA
ATGCTGGAGTGTACAGGTCTGATGGTCATATTGGCAACGAAGGGCAAAGATCTTCTTCAAATATTG
AAATTGAACATGGAAATGCTTACGGCAGTGGAAATGCAGGGTACTTAAGTGGAGCTAATGCTTACA
CAGGTGGTTCTCATACTTTAACAGCAGGAGCTGTCAATGTAGGACTTGTTGGAAAGACGACTTTAGT
CGATGCTAGCTACAACGATCAGTTTAATTATAATGCTGGTAATACTAAATCTCTCGGTCACGAAGTT
GTTCACCAATATAGCAACGGTCACGTTCCCGTCATTCAAAACTCAGGCGTTTCTATCAACCACATCG
GTACTGATCAAAACAATCTTGATGGCTACAGTTTCGTCACCACCCCAACATCATCTGGAATCCCTAC
TACAGCTACACCTTTTGCTGTCACTACCGCGATTCCAGTAACTACATACAAAACTGCATATGTACCT
GAAGCACCCAAAACTGACATCAAACAGATCTTTACGTATTCTCAACCTGCTGTTACCTACGTCCAAC
CAACGGTGTCCACTACACAGCAATTTGTCACACCTAAAGTCCAAGTTACAGATTATAACCAAGGGT
ACAATTACCAGGCTTCTAATAGCAAATCTGTTTCTTCAGGATACGTTTATTCCAAACCAGAAATCAA
ATTTGAAGATGGCGTAACTTATTCCACCCCAACACCGGTTGTAGTATCAACATTCAAACCTCAAGGA
TTTAGTCACACACAACAGACTGTTCACAACTTGGGATCTGGTTTCCAATATTCAACTCCTTTGCCTGT
CACGGAAGAACCTTTCAAGAAAGTAGTAGCTTACACCACTGGACCTGAGTTATCCTATGAACAACC
GATCGTTAGTTCTTACGCACCTTCATCTTTCAGCCAAGAAACAGTAAATAAAGTGCAAAGCAATAAT
GTCTACATTCCACCTTCTTACCAGTCCGTTACGTATGAACAACCTCAAACAGTTTTTGAACACACCA
CAGCGCAACCTGTCGTGGTAGCTAAATACCAACCCCAAGGTTTTAGTCACCAAACTTTACACAAAG
TCGATGCTAGTCAGGTCAATACGTATTCAGGCTCTGGATACAACTACCAGCAATCTTTGGACTCTGG
TTACAAATACGACAAACCTGCTTCTGGATATGATTACTTGAAACCCGAAATCAAGTTTGAAGAAAT
ACCTAACATACAATATTCGACTCCAGCACCCGTTGAGTCTACTTACAAGCCTTCGTCGTTCAGTCAT
CAAACCATTCATCAAGCTCAACATTACGTTCCGGTATCATCAACACCCGCTCCAGTGCAATTCACCT
ATCAACAGCCAGCTGTCACTTATAACGAGAACCCAATATTGAAGTACGTAGAAAACGTAACTCCTG
CAACTTACGTCCAACCGACTCCCCACTCTTTCAGTCACCAGACCATTCACAAAGTACAATCGAACCA
ATACGTTCCATCTGCCCCACTGGATACTGGTTACAAATATGAAGCACCTCAGACTGTAGTAGAACAT
ACAACTGCGCAACCTGTTGTTACAACAACATACCAGCCTCAGACTTACAGCCATGAAACGGTACAC
AAAGTTGAAGCAAGCAAAGTGAACACCTACAGTAACTCTGAATCAGGATACAGTTACGCACAACCC
GCTGTGAGATTTGAAGAGACTCCTAATATTCAGTACCCTACTCCAGCACCCGTAGTATCTACTTACC
AACCCCATTCTTACCATCAAGAGACTTACCATGAAACTAAACAATACGTCCCAGTTTCCACCCAAGC
CCCGAAAGTAAAAATTTACCAGCAACCTGCTGTAACTACCTATGAAAATCCCATCATAAAGTTCAC
GCAAAATGTTACTCCAGCACCTACTTACATACCTTCAGTTCAACAACATGAAATTAACCATGTTCAA
CAACAAAAACAGTATTACGACTACTCATACACTCAACCTGAAATCCAAAGAGAGTCTTTCCAAATT
TCTAATGCTGCCAAGTCCTACTCCGATGCTAGCCTCGTTTCTCATCAAACGCATCACCAATACAACA
CAAAGAACCAACAAGAATCCGGTAAAAATATAGTCTTCGTATCATCGACACCCTCGAACCTCATTT
ATGAAGACCATTACTCAGAATATGAAGCTCCCAAATTCAGAGCGCCAGAGTACGTACCACCAAAGA
CTTACTCTATATCGTCTACGGCTGCCCCAGTTATTCAACAAAGTTCAGTTTACCATCAAACCCAAGA
CCAATACGATTACAAACAAGGACAATATTCCCAAGACTCTAGTTCTTTGCAACAAGTAGCATTTAGT
TCTTCTCATGTCGAACAACCGAAAACTATAGAATCAGTTCATTTCTCATCTGTTCCTGAAATCCAGA
TTGAGCACAGAGAAGAATACGTACCAGTGAAAGTGGACAACGCTTACCTTCCCCCAGCAACTACTG
CTCGACCCATCGTTACTTCAACTTACAAACCAAGAACGTACCCTACTACTGAATACTTGCCTCCTTC
CACGAAAGCTCCAGAATATTTACCACCTCAACCTGAATACACTGTTCGTGATTATTTGCCCCCAAGA
GTTCCCACTTACCTTCCTCCTTCAACTACTGCCAGGCCATCTACCAAAGCCCCGGAATACTTGCCAC
CTTCAGAAGGCCATGTTGTCAATTACAATAGCTTTGGGTTCAACAACAACGACGAAAGCTACAACT
ATAACATAAATCCCTACCAACAAGTCACAGTTTCATCCACTCCTAAAACCAACTTGCTTGGTTTCGG
AACTGTTGGCCCTGATGCTGGATTAGTATCAACATTTACTACCGGTTCAGTGTCATCGACTTACTTG
CCACCGGTATCTTCAACTTATGCACCTCCATCTTCCTCATACATACCACCAGTGACATCCACTTACTT
ACCACCTCGTACAGCGAAACCCAGAGTAAGACCAACCACAAAAGCACCTGAATACCTACCACCTGT
TGAATCAACCACATTCAGAGCACCTGAATACTTACCTCCAGTAGACGAAAATGTCAGCTTTGGAAG
CTACGGTCTGAAGAATGCTCAAATCGCAGACAATCCTTACCAAGAACAAACAGACGTTTCTACATC
ATCCCCAGTTCAAAGGAAGCATAACATTGTTGTTCAAAATGCAAAGTCTAATTTGCTAGGTTTTGGA
ACGATCGGAAATAATGCCGGTCTTGTTACAGACACAGTGAGCTATTCTACTTCTACTCCAGTTACAT
CCACGTTTACTCCTGTCCAGCAGGCAGAAGTTCAAGTAACCCAAACACCTGCTAGAAGAACTAAAC
CGAAGGTAGCTGTTGTTACCAAAATTAACGATTTTAACCCTCTGTTGGTCAGAAAATTAGGAGCCGT
ATGCAGTTGCCAATCTCCTATTGTTGTACTAAAAGGAAGGAGACCTAGTGTCCAGGTACAGCAAGA
AGACTTTGATGAATATTCCGATGACACTGATTACCAAGATGGTCGTGGAGACATCAGGGGCAATGA
ATGGGCATTGAAATCTGGAAGATTGCAAGCTGTGAAGACGGTTACAGAATCTTCCTTCAATCCCATT

ATCGTTCCTGATGATTCCTACTACCAAGACGCCGATGAAATAGCTCCTAAGACTAAGCAGACTTATT
CTGAAGAATACGTCTCTAGTACCCCTGCATACTCGACAACTCAAAGAGTAGTGAAAATAAGACCAC
GAGTAAAGGCTGTGACCGTCGCTCCTACTTACAAAACTATTGTCTTGAATCAAGAAGTAGCTCCTAA
TGTTGTTGTCAAAGACACAGTAACAAGAGGCAGAGATGAGTTGCCTGACTGTCAAAGAGCTGGTCT
CTTCCGTCATCCAACAAAATGTAATAAATTCTACGCATGCAGGTGGGACTGCGACAAACAGAAGTT
TACCGTCCACACGTTCAACTGCCCTGTTCAACTAAGCTTTGACCCTAGTATAGGTGCTTGTAACTGG
CCAAGTCAAGGTCCGTCCTGCCAAGGTGATACTCTACTTACAAACTCGGTTTAA

> TaCht5 [organism=*Tuta absoluta*] chitinase 5 mRNA

ATGCGTGCAATACTAGCGACGTTGGCCGTTCTGGCGACATTGGCCGCTGCTGAATCGGATGGCAAA
GCCCGCATAGTATGCTACTTCAGCAACTGGGCGGTATACCGTCCAGGTGTGGGGCGGTACGGCATT
GAGGACATACCGGTGGACCTCTGCACGCACATCATCTACTCCTTCAGCGGCGTCACCGAGAAGTCG
CACCAGGTCCTCATCATTGACCCTGAGCTGGACGTAAACCAGAATGGGTTCCGCAACTTCACATCGC
TGCGGAAGTCGTACCCTGGTGTGAAGCTGATGGTGGCCGTGGGTGGCTGGGCGGAGGGAGGCTCCA
AGTACTCCCACATGGTCGCGCAGAAAAGCAGCCGCATGGCATTCGTATCCAGTGTCGTTGATTTCCT
GAAGAAGTACGACTTCGACGGCCTAGACCTGGACTGGGAGTACCCCGGAGCAGCAGACCGCGGAG
GCTCCTTCTCCGACAAGGACCGGTTCCTATACCTGGTGCAGGAGCTAAGGAGAGCCTTCATCAGGG
CGGAGCGCAACTGGGAGCTAACGGCTGCTGTGCCGCTGGCCAACTTCCGCCTCATGGAGGGCTACC
ACGTGCCCGAGCTGTGCGAGATCCTGGACGCTATTCACGTAATGTCGTACGATCTTCGTGGTAACTG
GGCCGGTTTCGCGGACGTACACTCCCCGTTATACAAACGACCTCACGACCAGTGGGCGTATGAGAA
ACTCAATGTGAACGACGGCCTCAACCTGTGGGAGGAGAAAGGTTGCCCCAGCAACAAGTTGGTGGT
GGGCATCCCGTTCTACGGGAGGTCGTTCACGCTGTCCCTGGGCAACCACAACTACAACTTGGGCAC
CTACATCGACAAGGAGGCTGGCGGCGGCTACCCCGCTCCCTACACCAACGCCTCGGGCTTCTGGGC
TTACTATGAGATTTGTTCAGAAGTGGACAGAGCAGGCTCCGAGTGGACAAAGAGTTGGGACCCGCA
CGGCATGTGTCCTTTCGCGTTCAAGGGCACGCAGTGGGTCGGGTACGAGGACAAGCACAGCGTGGA
GATCAAGATGAACTGGATCAAGAAGAAGGGCTATCTGGGCGCGATGACGTGGGCCGTCGACATGG
ACGACTTCCGAGGCCTGTGCGGCGACATGAACCCCCTGATGAAGCTGCTGCACGAACACATGAGCA
GTTACACTGTGCCGCCACCACGCACCGGGAACACTACACCCACTCCTGAATGGGCTCGTCCGCCATC
CACAACTCCTGACCCTCTGGAAGGCACGCCGGTGGTGATCCCCGGTTCCACAACCACCACGGCTGT
GACGGACACCACCATCGCTACACCCAAACCCACCACCACGGAAAGCCCTACCACACAAAAACCTGC
TGCTCCGGAACCCACCACATCGCAGGCAGCCCCAACAACTTCTGCACCATCACAGGAGGCTTCAGC
AGCTCCTGACGTAGAAGAGCCCCCCACGGATAACGAAGTGGAGAACCCTGACGTGTGTCTGAATGA
AGATGATTATGTCCCTGATAAGAAAAAATGTGACAGGTACTGGCGCTGCGTGAACGGCGAGGGTGT
GCAGTTCACGTGCCAGCCCGGCACCGTGTTCAACGTGAAGCTGAACGTGTGCGACTGGCCCGACAA
CGCCAACCGCAACGACTGCGCCGTCTGA

> TaCht6 [organism=*Tuta absoluta*] chitinase 6 mRNA

ATGTTCAGACAATTTGAACACGAATTACAATACCTACTCGCACTATCAACTCAGCCCCGCGTGGTAT
GCTACTACACAAACTGGTCGGTCTACCGGCCCGGCACCGCCAGGTTCAACCCTCAGAACATCAACC
CTTATCTCTGCACGCATCTCGTCTACGCCTTCGGAGGGTTCACCAAAGACAACACTCTGAAGCCCTT
CGATAAATACCAGGATATTGAGAAAGGTGGCTACGCGAAGTTCACGGGCCTGAAGACGTACAACA
AGCAGTTGAAGACGCTGCTGGCCATCGGCGGTTGGAACGAGGGTTCCTCCAGGTTCTCCCCCATGG
TCGCCTCCAGAGAGAGAAGGAAGGAGTTTGTCAGGAATGCCATCAAGTTCCTCAGACAGAACCACT
TCGACGGCCTAGACCTGGACTGGGAGTACCCGGCGTTCCGCGACGGTGGCAAGCCGAAAGACCGG
GAGCATTACGCCAAGCTAGTCCGGGAGCTACGCGAGGAGTTCGAGCGGGAGTCCGAGAAGACCGG
CAAAACTAGGCTGCTACTCACCATGGCGGTGCCAGCCGGTATCGAGTACATACAGAAGGGGTTCGA
TGTCAAGACTCTCAACAAGTACCTGGACTGGATGAACCTGCTCACCTACGACTACCACTCAGCATTC
GAGCCCGCAGTGAACCACCACGCCCCGCTCTACCCTCTCGAAGAGCCCAATGAGTACAGCGTGGAC
AATGAACTGAATATTGACTACACAATAAAATACTACCTGGAGAACGGCGCCGATCGTGACAAGCTA
GTACTGGGTATCCCTACATACGGCCGCTCCTACACGCTCTTCAATAAGGATGCTACAGAAATCGGA
GCACCTGCCGACGGCCCTGGTGAACAAGGGGACGCTACCAGAGAGAAAGGATATTTGGCTTACTAT
GAGATCTGCGAAGCTCTAAAACCAAAACAGCAAAAGAAACGCGCTGTCTCCGACGACGACTCCGA
AGAGGATGATTCCTCAGAAGAAGAAGTGGATGAACCCTGGACCGTCGTGCAGCCGAACCCCAAGG
CCATGGGTCCTTACGCGTACAGAGGGAACCAGTGGGTGGGGTACGATGATATAGACATTGTGAGGA
AGAAGGCGGAGTATGTGGCTGAGAATGGACTTGGAGGTATAATGTTCTGGTCGATTGACAACGACG
ACTTCCGCGGCACCTGTCATGGGAAACCCTACCCGCTCATTGAGGCTGCGAAGGAGGCTTACATCG
TCAAACTTGGTTCAACGGACAATTCCATCGTGACAGACAGGCCTCGATCCTCGACAGTTAGGAGCA
CCAGCAACAGAAGGAGAAACAGACCTCGCAGCTCAACTACAACCACTACCACTGAGAGAGCTGTT
GCTGCAAAATCGAGTAACAAGCGGAAGTCCGCCTCCTCAATCACCAGCACCACTCCGACTTGGAAC
ATCGTGACGCCTGAACCACCGACCACCCCTGACCCTGGATCTGACTTCAAGTGCACAGACGAGGGT
TTCTTCCCGCATCCCCGCGACTGTAAGAAGTACTTCTGGTGTCTGGACTCCGGGCCCTCCAACCTCG
GCATCGTCGCTCACCAATTTACTTGCCCATCAGGTCTATTCTTCAACAAGGCAGCAGATTCCTGCGA
CTTTGCTCGCAACGTGCTCTGCAAGAAGCCCGGCGCCACCACCAAGGCTCCCACTACCAAACCCAC
CGCAACCACCACCACTACCACTTCGACAACTACTAGGCAACCAGTCAAGCTGAGCACCAAGAGCGC
CTCGCTGTTTAGAACTACTACCACCACCACGACTACTCCTGAACCAGAGGAGGAGGAATATGAAGA
AGAGGCTGAAGATTCAGCAGAAATTGATGCAGAGGACCCTAAAGTTATTAAGGAGTTGATTGATCT
CATCAGAAAAGTTGGAGGTGTAGAGCAACTTGAGAAGCAGCTCCATCTATCGGAATCCTCGAGCAC
TGACGGTGCCATCACCACTACACCTGCGTCTATTGCTATCAACAAGCGACTTTACCAGAAGGTTCTA
GAAAGAACTATAGGAAAGAGCAACAAAATCAATGGCAACAGCGGCCCTGACGGTGGCAGTCCACT
AACAGCTCTCTACAGCAGAAGAGGTCCTCAGAACGCTGGACTCGAGCACACAGAGGATAAGGACA

AACTCCTGAAGAAGGATAGACCACAATACACAACAATTAACCGCTCTCGCCCTTCAACATCCACAC
AACAACCCGAATCAGAAGAAGACGAAGATGAAGACCTAGAAGACATTCCCGTGTCAGGACAGAGA
CAGACTCAAGCTAGAGTCGTAGAAGTGTCAAGTCAAGGAACTACAGCCAAACCTCTGCAGTACGTG
AACATAAGGAGAGCAAGACCACTGAGTACTTCCGACACAGCTGATGATTCTGCTTCTAGGAATGCC
CTGTTTGAGCGAGCTGAAACGTTCGTGACAGTGACAGAAACTGTCAACTCTTTGGACATAGCAAGC
GCACGCAGAGAGAACATTCCAGAATACGTGACTATAAGAAGGCAGAGGCCAACTACTGAAGAAGC
TACTACTGAACAATACCAGAACTCTGAACCGGAAGAGTCTCAAGAAACCGCCTTAGAAAGAGAGAT
TTCCTCACCCCAAATATCCTCTCAACCCCAATACATTTCTATAGCACGATTCCGATCCACTACAACC
ACAACTACTCCCATTCCTGAACAAAGTTCTCCTGAACCTACTACAGTCCTTGCCGTCCAAATATCTT
CCTTGTTGAATTCTCCTAGCTCCGAAGTCGAAACCTCGGAAGAGGATGCTAAAACGACTATCGATG
AAGCTACGGAAACTATCCCAGCAACTACACCGACGACCACAACTAGAGCACCTTCAAGTGCTGCTA
CTCGTCGAACAATTCTACGGAGAAGAGGCACAACAACCACCACCCAAGCTACTCCGTCATCATCCA
CCCAGGTGAGTTCAAGGAACTATTCTTTCGTTCGCCGTCGCCGCCCTATCGCCCAGCCAAATGAAAT
AGCTTCGGACTCAACAGAAGACTCTGAACTTTCTAGAAAAATTAGGTCTACCACTCCTGACAGTCGC
GAAACGGAAAGTGCTCGTACGGAATCCGTGCAGCCTCGCCGGGCTAGAGTTAGGTTCCAACAGACG
CAACAAGTTGATGACTCAGTACCTGCTGCTGCATCCCCTGTATCTCGAGGACGATTTCGGCCAGAGT
TTAGCCGCGATGAACTCATATCTCTGACTCCTGTTGACGTAGATATTCCAAGAGAATCTCCTCGATT
CGTTCCTAGGAGTTTTGGTTCTCGTAGCGAAAGTCGTTTCCGGGGTAGCACAACTGTATCCGATAAA
ACTGAAGATAGTGAAGTTTCAGCTGCTACTGTTGAGATAAGGCGACCAAATATTATACCTAGAGGT
CGAGGAAGATTTACGCCTAGTTCAACGACTGCAGCTTTAGATGAATCTCAAGTTTCCGAGTCGACGC
CAAGTCCTCGACGCCCAACGTTTGCTAGGTTCTCCCCGAGACCTTTTTCGAGAACAGTCTCAACACC
TGCAATAGACGATGAGGAATCAAAAGATATAGTTACTTCGAGGTTGCCAACTCGCCTACCTTTTGGC
AGAGCTAGAGTATTGACCTCAACAGCTGCTACAACAATATCATCTGTACCTGTACAGCCAAGGAGA
AACCTTTTCTCAGCCCGCCAATACAGTTCACCAAGACCAACATCACTTCAAAATGAAGAAGAAATT
GACCAAAACAAGGATGACATTGATCTTTCCGAAAGCACTGTAGCCGAACAAGGACATGAGGATAGT
ATAGACGCAGGAGATGAACTACCTAAACGAAGAGTAGTTATTAAAAAACACAGAACACCGGTAAG
TTCCACAACTGAAAGTACTGTTTCTACTGAAGCTGTACAAATTTCTGAGGACGGAAAAAAAAGAAT
TAGAGTTATTAAAAGGAGGCCAGCCTCAAGCACGCAAGCTACAGAGGAGCCAGTGCCAGAATCGA
ACGAAAATTCTTCTCCAGCACCACAAAGGATTCGAAAGGTTATTCGCAAAAAGATAAAGCTTGTTG
AAGATGAGCCAGAAATTATCGCTAAATCAATTGGCTCTTTAACATCCGCAAGAGACCTTACGACGG
AAATTGTTGGTAACTATGGCGAAAAAAGCCGGCCTGTATCTTCGACATTACTTACTCCTACAGAATT
TCCAGTTGAGAAAAGTACGGATGAAATTAATGAACCAGAAGCAACAAAAGAAGAAAACACAGAAG
TATCAAACGAATCAAACAATGATAAACCTGCAGAAGAAGAAAACAATAAACAAACTGAAGAATCT
AATTCTACAAACAGCAATAAAGAAGATGAATCTATCTCTAATACTGAATCAGAACCTAAGCCAGCG
GTGGCAGTAGAAACGGAGGTTAAAGCTATAGATTTGTCTGAAAATAATGAGAATAATGCTTCTGAG
GAACCGCAGCAAGACATTGAAGAAACTGCTCCGATTACACCTGCCACCACTACAACCATCAAACAA
CAGCCGTCGTCCACCCCTCGAGGTAGAGCTCCTTACAGACCTCCAAAGCGAACGTTCACTACTGCTA
CAGAATCATCTATACCTTCAAGTAGTAGAACATTTAGCCGTAAATTTAATCCAGGCGTATACACAAG
CCCCGCCACACCTGGGGAGAAAACTTCTCAGGCTACTACAAGAAAACCGTTTACATTCACAAGGCG
ACCTTTCACAAGAACAACAAAGAAAGTAGTAGAAGAGGAGGAGCTATATTCTGAAGAGGAGGAAG
ACTTTGTTGAACCAGAAGAAGAACATTCTCTAGTTATAGTACCGCCTAATCAACTGTTTAGTAAACG
ACCTAAACCTAGCGCAGAAACTGAAGAATCAGAACAAGTTTCTGGCGAAGAAGATGAAACAGAAG
AAGAACAACCACAAAGATTTATCCCAAGAAGACCGCCGACATTCCGTCCCAAAGCTGTTAATTCAA
ACACATTCAGAACGACATCATCTACAACTGAATTGCCTAAACGAAAGCAAAATAGAACAGCAATTT
ACACTAGATTCAGTGAAAATACAGAAACTAAGAAACGTGTGCAAAATGTTCCTTTTGGATACAGTC
CACCAGCTGTTAAGGCAGAGATTGATAATGAAGAGGACTCAAAAGAATATTTAACTACCACTTTGT
CGTTCACCGAAGAAACTGAGTCTACTACTGACGATGACTATCTGTCAATGACTGAAACTACTGGAA
GCTTTAATACGGCTGACGTAACTTCAACTAATACCGTAGACGGAGAAACAACTATGTCTGGTATAG
ATATTGATGATAGTACCGATGACTACTTGGAGTCAACGGAAGGTACAACTAACTTACCAACTACAC
AAGACTTTTACCTTCAAACTCAAACTGACAAAGAAACTACTTCTTATGAGCACATTACTACTTTTAA
TACTGAAGCTAGTCTTAATTCCGAAACTACTACAACAGAATCTATTCCTGAAACAACAACGGCGAC
GACGTCTACTACAACGACAACGACAACGACAACAACACCAGCTCCTGTTGTGAAAACACAGTATGA
TAAATTATTTTCAGTCAGCAGAGTAGTCGAAGTCAACTCCAAATTAGACAAACATCGCTTAAATAA
GAAAAACGAAACTACTTTAGTAGAAGAAGGGCAAATAATGGTAGAAAAGAGACCTACACTAGATA
AAATTGGAGAAGTAAGCAGGTTCAGTCTTATCAAAATTTATGAAGATGAAATACCAATTTATTTAA
CAAAATTAGGCCATGTTTATCCCGTAGAGAATCCACCGGACCATTTTATTCGCATTGATGAAGCAAG
GAATGCTAGAGCGCTAGTTAATTTTGCTGATGCTCCCAAAGAAAATTTAATCGCTTCAGAAAGTATG
AACGAAGCTTATAGACACATCAACAAAGTTACCAATCAACCTAAACATGAACCCAGTAGTAAAGGA
CTAGTAGAACATGTTAGTAATGATAATTTCTTGAAGTATATTAATGATGATAAGAAAAATAGCAAA
TCTGACGAGGATATATCGTTTACTCAGTGGCAATTCATTCCTGCCGCTTACGAAAATGAGCAAAATA
AAGGAGCTAAAAGCTTTGAAATAGTCACACCCAGAGGAATGCTCACTGAACCGTCTACTTTGCCAT
TGGAAGCACTTTTTAAAACTGAGAATCCTATGGCAAGAAAAGTTGACCAAAAAGGAGCCAACCAAC
CTTTCTTAGTATATTCTGCTTCATTGCCAAAAGAAGAAGCTAAACAATCATCAAAGACAGAGAATG
GTCAAAAAATTATCACATTTGCCAAAGGTCAAGAAATACACAGTGGTCCGGTTACAGAACAAGCAA
CAGTTAAATCCCCTATTGATATCCTTTCTCAAACGTCTCAAAGACCTTCATCAACAATAATTTCTATA
ATCTCAGACAGTACTAGTCCAATAATTGAAATGATGACAACTGCTTTACCTACAACATTAACAACTG
AACCATCCACTATTGCTTTAACAACAGATGCTGCTACGACAGAAACTGTTGTAGAAGAAACGACTA
CAACAAAGTCACCACTAGATACCAAAAGAACTAAGTTTGCCTTCCCCAGACGACCTGCATTCAAAC
CTACCAATGTGACTAGACCTACTATTGTGCCTCGGCTTGCCAAAAAGAACAATACATTGTCAGGAA

ACCTCAAAAAGAACAATACGTTGTCAGGAAATCTTTATCAAAAACCAAATAAGACTTCGACATTTA
ATGCAGCAAAGACAAGATACACTGGAAACAGATCACAAAATGTCCCAGTAGATATAAGAAAAAAG
ACCACAAAAGTGCCCATTAAGACTTTTACCACGGAAACGCCACGAACGACAACCGAAAGGAGATT
GTACGTAAAGCCATTCAGACCTAATTTGAGACCAGCATTTGTGCCTAGGAGAAATACGACACCATC
CACACAAACAATCACGGATACTTAA

> TaCht7 [organism=*Tuta absoluta*] chitinase 7 mRNA

ATGCACCTTCTACAGTGGCCTCCCAGATTGCTGCGGTGGACGGTCACCATCCTGCTGGTAGTAGCAG
TCCTTGCGCCCACCACAGATTCTACAAATGTCCGGCGAAGACTCCGGAAGCCCATCAAGTCCGTCTC
CACGTCAGTAACATCCAGCGTCTCCAGATCCTCAGACCAGATCATCTCAGCCAGCGTGAACAGACC
CAAGATCAGAGGCAGACCGAGTATTGCCAGCCGCAAGTCTTCAGCAGCCATCGACAACTCCGTCGG
CACTGAGGACCACAAGGACAAAGATGGCTACAAGATCGTCTGCTACTACACGAACTGGTCGCAATA
CCGGACTAAGATCGGCAAATTCCTCCCCGAGGACATCCAGCCGGACCTCTGCACCCACATCATCTTC
GCCTTCGGGTGGTTGAAGAAGGGCAAGCTCAGTTCTTTCGAGTCCAATGATGAGACGAAAGATGGC
AAGACTGGATTGTACGACAGAATCAACGCGTTGAAGAAGGCAAACCCTAAGTTGAAGACGCTTCTG
GCTATTGGTGGGTGGTCGTTCGGGACGCAGAAATTCAAGGACATGTCTGCAACGCGCTACGCCAGA
CAGACCTTCATCTACTCAGCCATCCCGTACCTCCGCGACAGGAACTTCGACGGTCTGGACGTGGACT
GGGAGTACCCCAAGGGTGGTGATGACAAGAAGAACTTCGTGCTGCTTTTGAAAGAACTCCGAGAAG
CCTTCGAGGCGGAAGCTCAAGAGGTGAAGAAACCTCGACTCCTCCTCACAGCTGCAGTGCCAGTCG
GACCTGACAACATCAAGAGCGGCTACGATGTACCTGCAGTCGCCAGCTACCTGGACTTCATCAACC
TGATGGCCTACGACTTCCACGGCAAGTGGGAGAGAGAGACAGGTCACAACGCGCCCCTCTACGCCC
CTTCGACCGACTCCGAGTGGAGGAAACAGCTGTCCGTAGACCATGCGTCTCATCTGTGGGTTAAGCT
TGGAGCGCCTAAAGAGAAACTTATCATTGGTATGCCAACATACGGGCGAACGTTCACACTGACCGA
CCCCAACCGATTCAAAGTGAATTCACCGGCTAAGGATGGAGGCAAAGCTGGCGAATACACCAAGG
AAGGAGGCTTCTTGGCTTACTACGAGGTGTGTGAGATGCTCCGCAGCCCTGGCGCCACCTACGTGTG
GGACGACGAAATGAAGGTGCCCTACGCCATCAAGGGAGACCAGTGGGTTGGCTTCGACGATGAGA
AGTCCATCAGGAACAAAATGAGGTGGATCAAAGACAACGGTTTCGGTGGTGCTATGGTGTGGACTG
TAGATATGGACGACTTTTCCGGTGACGTCTGCGGCGGGAACGTCAAGTACCCGCTCATTGGAGCTAT
GAGAGAGGAGCTCCGCGGCATCTCTCGTGGTAAAGACGCGAAGGACGTGGACTGGGCTTCTGTTGC
TGCCAACGTGCTGGTGGAAGTAGACAAGCCGGAGCCAGAGGTCATCAGTCTTCAGGAGGTTCTCAG
CAAGGTCAAGAAACCGCACAAGACCGTCATCGTCAAGAACAAGAACGCTGCCATTCTTGACAAGA
ATAAGCGCGAACCCCAAGTCCTGTGCTACCTGACATCCTGGTCTTCCAAGCGCCCCAGCGCCGGCC
GCTTCACTCCCGAGAATGTGGACCCCAATCTCTGCACACATGTCATCTACGCCTTTGCCACGCTCAA
GGACCATAAACTTTCTGAAGCTGATGAGAAAGACGCCGATATGTACGACAAGGTCATAGCGCTCAG
AGAGAAGAACCCGAATCTCAAGATCCTGCTGGCCATCGGTGGCTGGGCGTTCGGCTCCACCCCCTT
CAAAGAGCTCACCTCCAACGTTTTCCGTATGAACCAGTTCGTGTATGAAGCAATCGAGTTCTTAAGA
GACTACCAGTTCAACGGACTCGATGTCGACTGGGAGTACCCGAGAGGTGCCGATGACCGTGCTGCA
TTCGTCTCCCTCCTCAAGGAACTCCGCCTGGCCTTCGAAGGAGAAGCGAAGACCTCCGGCCAGCCC
CGACTCCTCCTCACCGCAGCCGTGCCCGCCTCGTTCGAAGCCATTGCTGCTGGTTACGACGTGCCTG
AGATCTCGAAGTACTTGGACTTTATCAACGTGATGACCTACGACTTCCACGGTCAATGGGAGCGCC
AAGTTGGACACAACAGCCCGCTCTTCCCTCTTGAGAGTGCCACTAGCTACCAGAAGAAACTCACTG
TGGACTACTCGGCTCGCGAGTGGGTCCGGCAAGGCGCTCCCAAAGAGAAGCTCATGATCGGGATGC
CGACTTACGGAAGATCATTCACGCTGATCAACGACACGCAATTCGACATCGGCGCCCCGGCTTCGG
GTGGTGGTAACGCTGGCCGCTACACCAACGAAGCAGGCTTCATGTCCTACTACGAGATCTGCGAGT
TCCTGCGCGAAGACAACACGACCTTGGTGTGGGACAACGAGCAGATGGTGCCCTTCGCGTACCGGG
AAGACCAGTGGGTTGGATTCGACGACGAGAGATCGCTCAAGACTAAGATGGCCTGGCTGAAGGAA
GAAGGCTTCGGCGGTATCATGGTGTGGTCCATCGACATGGACGACTTCAGGGGCTCCTGTGGAACC
GGCAAGTACCCGCTCATCACGGCTATGAAGCAGGAGCTCAGCGGGTACAAAGTTCGATTGGAGTAT
GACGGGCCCTATGAGTCGTCTAACCCCAACGGACAGTACACTACTAAGGACCCCAACGAAGTGACC
TGCGAAGAAGAAGACGGACACATCTCCTACCACAAGGACCGCGCCGACTGCACGATGTACTACATG
TGCGAGGGAGAGAGAAAGCACCACATGCCGTGCCCTTCCAACCTAGTGTTCAACCCTAACGAGAAC
GTCTGTGATTGGCCGGAGAACGTCGAGGGTTGCCAGCATCACACGCAAGCGCCTGCCGCCAGGCGA
TAG

> TaCht8 [organism=*Tuta absoluta*] chitinase 8 mRNA

ATGGGAGTGTTTTTTTATGTGCTAACGTTACTTTCTTTGAGTGCGTTTTATGTGGTTTCTAGTGAAAA
TAACGTAGTCTGTTACTACGGAACTTGGGCAACATACAGGCACGGTAACGGTAAATTCGACGTTCA
AAATGTGAATCCGTTCTTATGTACCCACCTCATTTACACGTTTGTGGGTATCGACAACCAGGGCAAT
GTTATATCTTTGGACTCTTACTTGGACCTGCCAGAAAATTGGGGAAGAGATAACTTTGGAAAATTCA
ATGCGCTAAAACAAAAGAATCCTAAATTGAAAACCTTGCTCGCTGTCGGCGGCTGGAACGAAGGGT
CCGCCAAATATTCCATTATGGCAGCTGACGCGAACCTACGCAAGAATTTCATCAGCTCAGCCCTGA
AAATGGTGCAGAACTACGGATTCGACGGTTTCGACCTCGACTGGGAGTACCCCAACAGACGGGACA
CGGTCCACGGCCAAGCTGACGTCAACAACTTCTCATTACTCGTTCAGGAATTGAAGGAGGAATTCTC
CAAATACGGTCTCCAAGTTACTGCTGCTGTCTCGTCGGTGGGGGACATGGCTTCGTTGTCCTATGAT
GTGCAGACTATTTGCCAACACCTAGACATCTTGAATCTAATGACCTACGATTTCTACGGATCCTGGG
ACACCACAACTGGTCACAACGCTGCTCTTCACATAGGACACAGGCATGGAAGTCCGTCGTTGTACG
CAGTGGACGGCGCTGTCGAGTACTGGCTCAGTCAAGGTTGTTCACCGGAGAAGTTGGCGGTTGGCG
TGCCGTTCTACGGAAGAACCTTCACCTTAGCAGACCCTTCCTTCAACTATGTGGGTGCTTCATCTAG
CGGTGCCGGTATTGCAGGGCCGTACACTGCCACTGCCGGAACCATAGGTTACAATGAGTTCTGCGT
CATGCTGCTAGCGGATTCAGGTTGGGAGGCCCGTTACGATGAAGAATCGGCCGTTCCATACGCCGT

CAATGGAAGAAACTGGGTCTCGTACGACGATGCAAACTCTATCACGAAAAAAGTCGAATGGGCTCT
CACTAAGAACGTTGGCGGTATCATGATTTGGAGTATCGAAACCGACGACTTTAACGGACTCTGTGG
CCAAGATTTTCCGTTACTTAGAGCTATAAATAAAGCATTGGGAAGGAATGTTGATGGCGATGATGT
AGAGGCTACGACTGTAGTCGAAGTTACTACAACACAAGGATCAACTGTAGAGCCTACGACGACGGA
TGCTACTACGGCAAAGGCTACGGCGACTGATGCTACGACGGAAGATGCTACGACGACAGCGGCTAC
GACAGATGCTACAACGGAAGAAGTATCATCACCACCTGACTCAGTGTGCGAAGCAGAAGGCATCA
GACCGAACCCTGCCAACTGTGCTTCGTTCCTCATGTGCATCAGAGACGCCAGCAGCCAATTGTACCC
GGTTGTGTTCAAGTGTCCCGCACAAACACTGTTCGACCCGGAGCATCTTTTCTGCGATTACAGTCAA
AATGTTCGCTGCAGAGTGTAATGAAACGAA

> TaCht10 [organism=*Tuta absoluta*] chitinase 10 mRNA

ATGACGTCGTTCTTACAGTGCGCACTGGCGCTAGTGTTGTGCGTACGCGCATCGCCGCACGCCGACT
ACAACACGTTCCTGCGCAGCGCGCCTGAGACCGTCCCGGATCATGAACCGCTCGGACCCCCCATCA
GATCCTCAGTCGAAAGCATTCCATTGAAGAGCCTTGGGGACGAATATGAAGGAGAGAAAAGACTG
CCGTTGCGAGATGCCGTCGAGAAGACGCCCATTCCAATATTGGATCCAGAAAATCTTTATAAAGCTT
TAGACGTTGACAAATGTCCCAAGTGTCGGAGACACAGCCCAACGGAAGGTGGTGTGCTACATGCAG
TCCCTGGCGGCGTACCGCGCGCCTCCGCTGGCCTTCACCGCCGGTCTGGTGCCAAGATCTGCACACA
TCTTCACTATGCTTTTGCGGTGATTCATCCGCACACGTTTGCCATCTTACCCGCGAATGAGGACTATG
ATCTCGTCCGAGGAGGTTATCGCATCGCGACGGGTTTGAAGAAGCGTTACCCCGGTCTGCAAGTGA
TGTTGAGCGTGGGCGGCGACGGGACAGACCGTCTGTTTAGCGACATGGTTCAGGAGACGAGAAGAC
GTAGCAACTTCATAGAGAGCGCAGTGGCCTTCTTGCGGGAGCATGACTTTGATGGACTTGATCTCCA
TTGGGTATACCCAGGCGATGATGACCGGGAAAAAGAAATGCTGACCACTCTTCTGTACGAGCTGCG
TGAGAAGTTCTCAGCCTATGGACTCCTACTTCAACTGTATTGCCTCCTTTCAGTGAGTGGTGCGACG
GACTACGTGGTACTGCAGGCTTGGGACATGACGCATGGCAAGCGCGACGAGCCGCCACCAAGGGC
GGTGCAGCACAGCGCGCTGCATCGAGACCCTGGGGCAGCTGCCAGAGATCAGAGATACGATAACA
TTGAGTTTATGGTGAAATACATAGTGCGCCATGGAATGTCAGCCGACAAACTGGTGTTGGGAGTGC
CTGTTTTCGGCCGAAGCTACAACTTGGCGGCATCTACACTACCGTCGCCAGGAGCATTGGTCTCTGG
CTGGGGCGATGAAGGGCAGTATACGCAAACGAAGGGGCTGCTTGCCTATTTTGAGATTTGCATGAT
TGAACGTGAAGGCAAAGGATCGACTGGTATCGACGAAGCCGGGAACTCCTACGCTGTTTTTGACAA
CCAATGGATCACTTACGATACGCCTTCTAATGTTTTGGAAAAGATGAAGTTCGTAATCAGCACTGGA
TTGGCTGGCGCGGCAGCCTGGGCTATTGACATGGATGATTTCCGCGGACTTTGCGGCACACCATTCC
CTATTTTGAGTGCTATTGCCACAAGTCTTAACGGAGAGACTCTACAGACCGACCAGTCATCGCTGAA
GCTGGGCGAGTGTGACCCCGGCGACCCTTACCTGTCCTCGGACGAGCATTCCTGTTCACACTTCCAG
TTCTGCTCGGGAGGTGTCAACTACCGGCTGGTCTGCGAGGACGAGCGACTCTACGACCCTTCCACTG
GATTCTGCGGGCATCAAGATGTAACGAAATGTGTTCCCGGACAGAACCTAAGAATCAACGTGGCAG
ATGCGACGCGGTATTTAAGTCAACCGGACGAGGCTGACTTCGAATGGGGAGATGATCCGTTGAAAG
AAGTCATCAAGAAGACTAACGAAAGAGAGAGGCTTGTCGATGATGCAGATTATGACACCAAGAGA
ACCAAGGCGAACGACAAACGCGTGATCTGCTACATGACCAGCTGGGCGTTCTACCGGAGGGCGGAT
GGCAAGTTCGTCCCGGAGAATATTGACACGCGCCTCTGCACGCACGTCGTCTACGCATACGCCTCAC
TCTCGCCCAGCGATCTCATCGCCAAGGAGTTCGATCCCTGGACTGATATCACTAACAATCTTTACGA
GCGTGTGACGTCCCTCAGCGACGTGAAGGTGCTTCTTGGTCTAGGCGGATGGACAGACTCAGCCGG
CGACAAATATTCTCGCCTTGTGTCCTCCCCTTCAGCCCGGGCCAAGTTCACCGAGAACCTGGTGTCT
TTCCTGCGCATGCACAACTTTCAAGGGTTGCATCTGGACTGGAGCTACCCCGTGTGTTGGCAGAGCA
ACTGCAAGAAGGGCGCTATGTCGGATAAGACTAACTTCGCCAAATTGGTTCAGGAACTTTCAACAG
CTCTACATAAAGCAAATATGGAGATAGGCGTTGCTATATCTGGATATAAGGAGGTCATCGAAACTG
CTTACGATTTACCTACATTGTCCAAAGCAGCAGACTTCTTGAGCGCCATGACTTACGATTACCACGG
CGGGTGGGAGCGAACTACCGCACATCATACACCTCTTACACCCTCTTCGAAGGACAACTTGCCATA
CTACTCTATTGAATACGCTATAAAGGCAATGATAAGCGGCGGTGCAGACCCGAAGAAGTTGAACCT
TGGTCTTTCGTTCTACGGCCAATCGTACAGACTTCAAATGGCGGAAGCCACAAGTGGTCCAGGAGC
TCCTGCCTCCGGCCCTGGTGAACCTGGAGAGTTCACTAAGCAGCCAGGAATGTTAGCGTATTACGA
AATATGCTACAGGGTGAAGATGTTGCGCTGGAAGACAGGTCGTCAAGAAAAAGCTGGACCATACG
CATACTCTGATAACCAGTGGGTTGGCTATGATGATCCTAAGTCGATTGCTGAAAAGGTGGAATGGG
CGCTAAGCCAAGGTTTAGGTGGTGTCGTAGCTTGGGCAATAGACTTGGACGACTTCAGCAACCGGT
GCTGTGCGGAGCCATCGCCGTTGCTGCGTGCTGCTGGCCGAGCGCTAGGGCGTTCCGTGCCGCCACC
CCCGCAGTCTCCCTGCGAACGGCCGCCGCCCCCGGTCACCCCCGCACCACCCACTACACCACCTCCT
GCAGAGTCTGATGGTTCGCTAAGTAGCACAACCCCAAGTCACCACCACGAGCACCATACGACTTCA
ACAACCACGCAATGGTCCTGGAACCCTGCCACCACCAGCCCGACTACTTCACAAACCTGGTGGGCT
CCAGCCACTACGACGCCCATGACCACCACCACCACTAAAGCAACCACCACTTCGACTACTCGAAGA
CCCACTGCAGCGCCATCCAGACCTCCAACTGGTGACACAGGAAGCTTGGAGGGCACATCATGCGTG
GCAGGAGAATATCGCTCAGCTCCAGAAGACTGTGAAGGTTACCTGCAGTGTGAGGGAGGGCAATG
GCGCAAGCACCGTTGTGCTCCAGGGCTCCACTGGTCTTCAAGCGCAACCCGTTGTGATTGGCCGAGC
TTTGCCAAGTGCAGAGAATCACCGAGCGAAGCAAGCACCGCGACGACGACTCTCGCGCCGATGACG
TCTCGGCCGCCACGTCCTGCCACTACGACTACAACTACTACCACCACCAGACGCACCACCACATCTA
CTACCACCACTGCTAGACCAACTACTTCATCGACAGCAGCTGCTTCGGGTGATCCATCAGTAGGCAG
AGCCTGCAATAGCCAGCAATACCAGCCGGTGCGAGGTGACTGCAACTCGTATCTGCACTGCGACGG
CAACGTGTGGCGACTCCAGCACTGTGCTCCTGGTCTACACTGGAGCCAAGTTGCCACTCACTGTGAC
TGGCCCAAATATGCCAAGTGTCAAGGTTCTGAACCACAAACTCCGACTTCAACATCGAGACCGCCA
AAGCCGCCTACAAGACCGCCAAGACCAACTAGGCCAACCACTGTCTCACAAGCCGACAGACCAGTC
GAAGATGGGGAGTGCGGAGGCAACGAAATGCATGCAGCAGCATCGTCGTGTGACGCCTACCTGCTC
TGTGTGTCGGGCCGCTGGCGCAAACAGCTGTGTCCACCAGGCTTGCACTGGGACCGACGCAGCAAC

CGCTGCGACTGGGCAGACTTTGCCATGTGCGAAGCATCTAAACCTGACGCGACAAAATCTCCAGTA
TCTGCAACCAGCACAACACGACCAGCCGCAACCACGTCTAGAAGACCCACGTATACAACAACCAAA
AAGCCAAAACCTATTATCGAAGACGTCGACCCTCTGAAACCTGGAGAGCGTTGCCAAACGGGAGCC
TACTACGCCCACCCGAAATGCGAGAAGTTCTTCGTATGCGTGAACGGCATGCTGATCGCCCAGAGC
TGCGCCCCGGGGCTGGTTTGGAATGCCCAGCGCTCCCAGTGCGACTTCCCAACTGCCACCGCTTGTT
CTGATAGGAGACAGGTTTCTGCTGCTATGGTAGATGCCAGCAAACCAGACACCAGCCATGATGAAC
AAGTTGAAGAATATTGCAACAACGGAGAATACGCGAAGTTGGAATCTGATTGCACACGGTACAGAC
ATTGTATCTTTGGCAAGTTCCAAGAATTCGCCTGCAGTCCTGGATTACACTGGAATCAGGAGAAATC
AATATGCGATTGGCCCAAAAGCGCTAAATGCAAAGCAAGTGTCCCAATGAAACCTGCGGTACCAGC
TAAACCAGTCTCACCACCAAAGACAACAACATCAAAGCCTACACCCCAGCCACAACCTGCAGAGGA
AAATTATCCCGAGCAGGGTCATCACGAAACTCATCCGCATCCAACAAAGCCAGTGGGAGCAGTACC
AGGACCCGGTACCTCAGTTCAAGCTTCCAAACCACAGCTTTTGAATACACGATACAAGCTGGTATG
CTATTACACGAACTGGTCTTGGTACCGACCGGGTCTGGGCAAGTACAGTCCGGAGGACATTGACCC
TACTCTATGCACACACATTGTGTACGGATTCGCAGTGTTAGGCAATGACGGTCTCATCACGGCGCAT
GACTCTTGGGCGGATTATGACAACCGTTTTTACGAACGAGTTGTAGAGTACAAGAGATACGGTATT
AAGGTGTCAATCGCCCTCGGTGGATGGAATGATTCAGTTGGCGACAAGTACTCTAAATTGGTGAAT
GATCCTGCAGCGCGTGCTCGCTTTGTCGATCATGCTCTGGAATTCGTTGAGCAGTATGGATTCGACG
GCTTGGATCTGGATTGGGAATATCCCAAGTGCTGGCAGGTTGACTGCTCTAAAGGTCCCGAGTCCG
ACAAGTACAATTTCGCAAACTTAGTTCGTGAACTATCTGCTGCATTCAAACCCAGGGGCTTGTTGTT
ATCTTCTGCTGTATCGCCGAGCAAAAAAGTCATAGACGAAGGTTACGATGTACCAGTTCTCGCAAA
ATATTTGGATTGGATCGCAGTCATGACGTACGACTACCACGGACAATGGGATAAGAAGACGGGTCA
TGTAGCGCCGTTGTTTTACCACCCTGATGATGATTATACTTACTTCAATGCCAACTACACAATGCAC
TACTGGATGCAGAAAGGAGCGCCATCCTCCAAGCTTATCATGGGAATTCCATTGTACGGTCAAGCA
TTTACTTTAGAGAACCAAGTGGGAAGTATTAGAGACTTTGGAATGGGTTTAAACCTCCCTGCTGTGT
CTGGTGGCGAAGCTGGAGAGTACACGAGAGCTAAAGGATTTTTAGCTTACTATGAGATTTGTGACC
GAATCAGAAACCAAGGCTGGAAGGTTGTACGTGATTCACAGCAACGTATGGGTCCGTATGCATTCA
AAGACAATCAGTGGGTCTCGTTTGACGATCAAGAGATTATCAAAAAGAAAGTCAACTTCATCAAAT
CTTTAGATTTGGCTGGTGGTATGGTTTGGGCCATGGATCTGGATGACTTTAGAAACAGATGCGGGCA
AGGAAAACACCCTCTACTGAATACAATTAAGGACGGACTTCTAGATCCGAATACCGAGTTTGAATC
TGCACAAACTGAACCTGTACTTGGACCACCAAATGAAATAGATGTAGATTTGGAAGACATCGAAGT
GAGGCCAAGTTACACCAAGCCTAGCACAACTCCGAGGCCAATGAAAATGCCCACGAGGCCACAGG
CAACACAACCAACGACTACGAAGAGACCACAAATTACGGGCTCATTTAAAGAAGAGCGCTTTAAA
ATAGTCTGTTATTATACCAACTGGGCTTGGTACAGACCAGAATCGGGCAAGTACTCACCAAGCGAC
ATTGACCCATCTCTTTGCACACACATCGTGTATGCGTTTGCAGTTCTTGACAACAACAAATTGGTCA
TCAAACCACACGACATTTCACTGGACATTGAAAACAAATTCTACGAGAAAGTAATAGCGCTTAGAA
GCAAAGGAGTGAGAGTCCTCCTTGGTTTGGGAGGCTGGAATGACTCTGCTGGCGATAAATACTCTC
GGTTGGTAAACAATCCATCTGCAAGACGGAAGTTCATTGTCCATGCTTTGGACTTCCTGGATCAATA
CGGATTCGATGGTTTAGATCTTGATTGGGAGTACCCAAGATGCTGGCAGGTAAACTGCGAAAAAGG
ACCAAGCTCTGACAAACAAGGATTTTCATATCTTGTGAAAGAACTCCGTGCAGCTTTTGAACCACGT
GGTCTACTTTTGTCAGCCGCAGTTTCTGCCAGCAAACGAGTCGTCGACCTTGCATATGACGTTCATA
CCCTATCGCAGAACCTAGACTGGATTGCATTGATGACATACGATTATCACGGACAGTGGGATAAAA
AGACCGGTCATGTATCACCAATGTACGCCCAGGAACGTGAAGATATGACTTTGAATGTGAACTACA
CAGTTCACTACTGGATGAGTAAAGGCGCATCACCAAAGAAACTGGTTATGGGAATGCCGTTTTATG
GACAATCCTTCGCCTTGGCAGAACATGCCGGAAATGGATTAGGTGCGCCAAGCTACGCTGGTGGGG
AAGCCGGGGACGAAACTAGGGCTAGAGGCTTTTTATCGTTTTACGAGATATGTGATCGCATCCGAT
CTCAAGGCTGGAAGGTCTATCGCGATCCAGGCGGTCGTATGGGACCGTACGCAACTCGTGGCGACC
AATGGGTGTCATTCGATGACGACTTCATGATCCGCCACAAGGCTGAATACGTTCGCTCCATGGAGCT
AGGAGGCAGTATGGTTTGGGCGTTGGATTTGGATGACTTCACTGGAGAACATTGTGGTTGTGGAAA
AGCACCTCTACTGCGAACTATTAACCACGTACTCAGAGAAACTATGGCACCGCCACCTTGTTCTCTT
AAAGAAATTGAAGCTCCGCAATCGCCAGAAGAAGTATCAAATATAGAATCACCTGTGCCATCGGAA
CCCGACACAGTACCATCAGAACCTGAATTTATTCAACCAGAATCAGAAACAGGACCTGAAGACGAT
TCACAAAATCAAAGCCCGACTTTGGAAGGACAGTCATGTCAAGAAAGCACATTTAAAGCAGATAGC
ACAGACTGCAGAAAGTACTATTTGTGTATAAACGGCCAATACATACAACTAACTTGTCCAGAAAAG
CTGCATTGGAACAAGAATCATTGTGACTGGCCATCAAGATCAAATTGCAAAATTAGATCCAACCTT
AGATTGACAGAAGATCAAGAAAATAATCATGGAACAGTTGGGACCCAGGAATTAATTGAGCCCATT
GTAGGTTGCTACTTTACAAACTGGGCGTATTACAGGCATGGCACAGGAAGCTTTGGGCCGGCTCAA
TCGGACTTCTCACTATGCACGCACGTAATTTATGCATGGGCGCATTTAAATAGCACCACATATAAAA
TAATTCCTGGTAACCACGAATTAGATATTGACAATGATTTCTTCGGAAAAATATCTGAGCTGCGTAA
AAAGGAAGTGAAACTCATTCTCGGAGTAGGAGGTTTAGAAGACTCTGAAGACAAGAAATGGAGCC
GCATGGCTTCAAACCGCAACCACAGGAGTGCTTTCATTAGCTCTGTTCTAAAGTTCCTCAAACGATG
GGACTTTGATGGGTTGCAGTTAGCCTGGCAATATCCCGTGTGTAAACAGATTCCGTGCGACGAAGA
CAGATTATTTGACAGAGATCACTTCAGCACACTGCTATTGGAACTATCTGGAGCTCTACGCTCTCAT
AACCTGGAGTTTTCAGCCATGGTAGCTGCGGCTCCTGAAGTAGCTGCGGAGGCGTACGACCCGAGT
GTATTAGCGCATACACTGGACTGGCTGTCCATAGCAGCAAATGATTACTACGCTTCTACAAGCGGG
AAGACCGCGTACTTGGTGCCATTGGAAACCCCTGAAGCAGCGGGAATTAATAGCTTTAACTCGACT
CTAGCCTTCTGGGCGAGCGCGGTGCCAACACGACAGCTGGTGATAGGAATACCAGCATACGCAAGA
TCGTACACATTGCGAAGACCTGATGCATCCGGCCCTGGAGCAGTAGTCACCGCGCCCGGAAGCCCA
GGGCCTTACACAGGAATACCTGGCTTCCTTGCGTATTATGAGGTGTGCTCCGAAAGTGGAAAAGCA
AAGAAGTGGCACGAAACTATCACCGAGGACGGAACCTATGCTATAAGTGGTAACCAATGGGCGTCC

TACCTTCGCCGAGAAGAGGTGCACCGGGTAGCGTCGTCGGCGTCGCGCGCTGGTCTCCGGGGGGCT
GCGCTCTGGGCCATGGATCTGGACGACTGGCGCGGCGACTGCGGGTGCTCCAGGCCGCTCCTCACA
GCGATGTGGCAGGGCCTCGTCAACCCCGACGTAGCGCCCTCCTGCCTGCCATAA

> TaCht11 [organism=Tuta absoluta] chitinase 11 mRNA

ATGTATCTACAAATTAGTTCGGAATCTGCAACGAAAATGGGTTACGTTGATCTAGATGGCCAGCAC
CAAGTGAGTTCAAGCAGGAGACGGCAGGCGTGTGCGGTGTGGTGTTCAATAGCGGCGATGGTGGCG
GTGACGGTGGCGGGGGTGAGCCTGCTGGGAACGGCCCGACGCGCGTGGCAGCTGGAGGCATCAGC
ACGCGGAGTGGGAGCGGGGGGAGTCCTCGTGTGCTACTACACGGCGTCGCAGGCGCTGCAGCCGCG
CGACATCCACCCGCAACTCTGCACGCACATCAACGTCGCATTCGCGCAGGTTCAGAACAAGCAAAT
CAAACTAGACGAAAATCTACACAAAACTCTCCTAGAAGTTGTCAAACTCAAAACTATCAATCCATC
CCTAAAGATCCTACTTTCTATTGGAGGAGCAGGAGATAATGATGGTTTTTCTGACATGGTAGTAGAC
CATGCATCCAGGAAGACATTCATTAGGTCCATTAAATATGTTCTAAGAAACTATAACTTGGATGGA
ATTGATTTGGACTGGGAATTTCCTGCTGTTCATTATGATGGGATTAAAGGAAAACGAGAGCGTCAG
CACTTCTCTCAACTGCTCCGAGAGATCAGAGCAGAGTATGTCCGGGAGAAGCGGAGCTACCTGCTC
ACTGTTGCCGTAGCGGCGCAACAGAGCATTGCAGACACCGCCTACGATGTTGACCAGATGAACATG
TATGCAGACTTAGTCAATATCATGACTTATGATTTTCATTTCTTTACTAAGTACACTCCATTTACAGG
ATTGAATTCTCCATTATATGCAAGAACTTCTGAAGAACTATTTTTTGCTTCATTGAACATTAACTTTA
CTGTAAATATGTATTTAGATAAAGGTCTTTCAAAAGATAAACTGGTAGTGGGCATACCAACATATG
GTCACACATTCACACTTGTGAATGCAGACAATATAGGCATAGGAAGCCCCGCTTCAGGCTTTGGTTC
TCTTGGCTCACTTGGTTTTGTGAACTTTCCGGACATTTGCATTTTCATACAAAATACCACCAATAAAG
TAGTGGTGAAACAAGACAACGATGCCAAAGTTCCATACCTGAGTAGTGGTTCAGAGTGGGTGTCAT
ATGACTCCCCGCAGAGTGTCACAGAGAAAGCAAAGTTTATAAGATCTAATGGTTTGAGAGGGGCCA
TGATCTACTCTCTAAATGCGGATGACTACCAGGGAGTGTGCAGACAAGGTGTGGGCAATGACATCA
AGTTCCCTCTTGCAAATGCTGTCAGGAAACTGTTAACTGATGATGAAAGTTTGTAG

> TaCht-h [organism=*Tuta absoluta*] chitinase gene mRNA

ATGAGGCACCAGACGTTGGCGCTGCTCGTCGGAGTGGGGCTTCTAGCCTTAGCCCACTGCGCGCCTC
CTGGGAAGCCCCAGCTCGGTTGGGGTGAGCGCACCTTCGCCATAGTGGAAGTCAACCAGGCAGCTA
CGGCATACAATCAGCTCGTCAAAAGACACGATGGCGCCGATGTGTCTGTTACGTGGAATGTATGGT
CCGGTGACCCAGCTACCAGCTCCAAGGTGCTTCTCGACGGTAAAGAGTATTGGTCTGGAAATGGAG
GCTCTTCTGGCGCAGCCCATTTCAAAGTAAGGAAGGGCGGAAGATACCAAATGGTGATAGAGCTGT
GCAATAGTGATGGATGCAGTCATAGTGAAGCAACTGAGATTGTTGTGGCTGACACCGACGGTAGTC
ACTTGCCTCCGCTAGAATACTCTCTTGGTGAGAAAAATAAACCATTTAAACAAACATCAGGAAAAG
TGGTTGGTGCTTACTTCGTTGAATGGGGTGTGTACCCTAGGAAGTTTCCAGTAGATCGAGTGCCGGT
TCCCAACCTGACTCATTTATTGTATGGATTTATTCCGATTTGCGGTGGAGATGGTATTAATGACAGC
TTAAAAGAAATTGAAGGAAGTTTTCAAGCACTCCAACGTTCTTGCAGTGGCCGCGAAGCATTCAAA
GTATCTATTCATGACCCTTGGGCAGCTTTGCAAAAGCCACAGAAAGGCTTGTCATCGTGGAATGAA
CCATACAAGGGTAACTTTGGCCAACTAATGTCCCTGAAACAAGCCAAACCTGATCTGAAAATCCTT
CCTTCTATAGGTGGCTGGACATTGGCAGACCCATTCTATTTCTTTGATGATTCAGTCAAACGGCACC
GCTTTGTCGATTCAGTTAAGGAATTCTTGCAGACATGGAAATTCTTTGATGGGGTAGATATTGACTG
GGAATTCCCTGGAGGTAAAGGTGCCAATCCTCATCTTGGGGGTCCTAACGATGGACACACATACGT
TACATTGATGAAGGAGCTGCGTGAAATGCTAAATGAGCTCTCCGCTGAAACTGGCAAGACCTACGA
ATTAACTTCAGCTATCAGCGCAGGTTGGGATAAGATTCAGGTAGTGGATTACAGTGCTGCACAAAA
ATACATGGATCATATATTCCTGATGAGCTACGACTTCAAAGGCGCGTGGTCTAATGACACGCTTGGC
CACCAGACGCCTTTGTACGCTCCCGCATGGAGGCCAAAAGAAACCTACACTACTGACTTCGGAGTT
AAGTATCTGTTAGCCCAAGGAGTGAATCCTAAAAAGATAGTTATCGGTGTAGCTATGTACGGCCGC
GGTTGGACTGGAGTACATGGATACGAAGATCGTTCTAATCCTTTTACTGGTAATGCAACTGGTCCAG
TCAAAGGTACCTGGCAAGATGGTGTGGTGGATTATAGAGAAATTGCTAATGAAATAGCAGCAGGCA
AATGGGAATATAAGTATGACAGTACAGCACAAGCACCTTACGTGTTCAGACCGTCCTCGGGAGACC
TTATTACGTATGACGACGCCAGATCGGTCATTGCGAAGGGCAAGTACGTTAGGAATAATAAACTAG
GAGGTCTATTCGCTTGGGAAATAGATGCAGACAACGGCGAACTACTCAATGCTATGAATATGGGTC
TCGGAAACAGTGCATCGTGAAA

> TaIDGF [organism=*Tuta absoluta*] imaginal disc growth factor protein (IDGF) mRNA

ATGAAGAGCCTGATTGCTTTCCTTGGGGTGTTGGCGGTAGTGGCCGCCAACCCCACGCCCGCCACCG
GCAAGGTTGTCTGCTATTACGACAGCAAAAGCTATGTCAGGGAATCTCAAGCCCGCATGCTGCCTCT
GGACTTGGACCCTGCTCTGTCTTTCTGCACTCACCTCGTCTACGGATATGCTGGAATCCAACCTGAC
ACCTTCAAGATGGTTCCTCTCAACGAGAACCTGGACGTAGACCGGTCGCACGCCAACTACAGAGCC
ATCACCAACTTCAAGACCAAATACCCTGGTCTGAAGGTCCTGCTCTCCGTCGGAGGTGATGCCGATA
ACGAAGAAGCACAGAAATATAATCTCCTGCTGGAGGCACCGCAAGCGCGCACCGCTTTCGTCAACT
CTGGAGTCCTTCTGGCTGAGCAGCACGGCTTCGACGGCATCGACCTGGCCTGGCAGTTCCCCAGGAT
CAAGCCCAAGAAGATACGCTCTACTTTTGGGTCCATCTGGCACGGTATCAAGAAGACCTTCGGCTC
GACCCCCGTGGACGACAAGGAGGCGGAGCATCGCGAGGGGTACACGGCGCTCGTGCGTGAGCTGA
AGCAGGCGCTCAGCGTCAAGCCCAACATGCAGCTCGCCATCTCCGTGCTGCCCAATGTCAACGCTTC
CATCTACTACGACGTGCCCGCTATCATCAACTTGGTGGATATCGTGAACATCGACGCGTTCGACTAC
AACACTCCCGAGCGCAACCCCAAGGAGGCCGACTACGCGTCCCCCATCTACACGCCGCAGAACCGC
AACCCTCTGCTCAACGCTGACGCTGCCGTCAACTACTGGCTGCAAGCTGGTGCCCCTGCCAACAAGC
TAGTGCTGGGCGTGTCCACGTTCGGGCGCACCTGGAAGCTTGACGCCGAGAGCGAGATCGCCGGAG
TCCCCCCGCTGCACGCCGACGGACCCGGCGAGGCTGGTCCTTACACCAAGACTGAAGGCATCCTGA
GCTACCCCGAGGTTTGCGCCAAGCTAATCAACCCCAACCACCAGAAGGGCATGAAGCCACACCTCA

GGAAGGTCACCGACCCTAGCCACAGATTCGGAACTTACGCGTTCCGCCTGCCTGACGACAACGGCG
AGCCCGGCCTGTGGGTCAGCTACGAGGACCCCGACACAGCGGGACAAAAGGCCGCCTATGTCAAGT
CAAAGAACCTGGGTGGTGTGTCTATCACCGACCTGTCAATGGACGACTTCCGTGGCCTGTGCACCG
GTGACAAGTACCCTATTCTGCGCGCTGCAAAATACCGCCTCTAA
