# Supplementary material for: Identification and Functional Characterization of Chitinase Genes During Larva–Pupa–Adult Transitions in Tuta absoluta
Source: Insects. 2026 Jan 20;17(1):114. doi: 10.3390/insects17010114 (PMC12841776; doi:10.3390/insects17010114)
Supplement: Supplementary file 1 [file insects-17-00114-s001.zip › Table S2.pdf]

Table S2. Primers used in this study.

Appilation primers	Gene name	Forward primer (5′-3′)	Reverse primer (5′-3′)
Full-length clone	*TaCht1*	AGATGCAATGTCTGAAGAC	ATTCCGGTTCCATACTCG
	TaCht2	TTTTGACCGTGGCTTCTG	AGTTTGCCGTTTGATTGC
	TaCht3	ATGTGGCTTTGGTTGTTA	CTCAGGTCCAGTGGTGTA
	TaCht5	TACGACAAACCTGCTTCT	TTGTTGGATGACGGAAGA
	TaCht6	TACAATACCTACTCGCAC	CTTTAGGGTCCTCTGCAT
		ACAACCCGAATCAGAAG	TCCGTTTCTACTGCCACC
		GAAACTGCTCCGATTACA	CACAAATGCTGGTCTCA
	TaCht7	CGCCCACCACAGATTCTACAA	TTCTCCGGCCAATCACAGA
	TaCht8	CGGAACTTGGGCAACATA	TTGTAGCATCTGTCGTAGC
	TaCht10	CCTAGACCAAGAAGCACC	CCTAGACCAAGAAGCACC
		TGGAGATAGGCGTTGCTA	CGTAGTAGGCTCCCGTTT
		GGCAAGTTCCAAGAATTC	ATTTATCGCCAGCAGAGT
		TTTCTGCCAGCAAACGAG	ATAGTTTCGTGCCACTTC
	TaCht11	TTAGTCGCTGGGTCAAGG	CTTCAATACTGGTCATGG
	TaCht-h	AAGTGAAGTTTGCGTCCA	GATCTGGCGTCGTCATAC
	TaIDGF	GAGCCTGATTGCTTTCCT	TCGTGGCGTGGACTCTAT
qPCR analysis	*TaCht1*	GTCCGATAAGAAGGGGCAGA	ATTCCGAGAAGCCACATCCT
	TaCht2	GTTTTGACCGTGGCTTCTGT	CATGTGGATGAGGGTCAGGT
	TaCht3	GCCTTCGTCGTTCAGTCATC	TGGGGCAGATGGAACGTATT
	TaCht5	AGAAGAAGGGCTATCTGGGC	AGGGTCAGGAGTTGTGGATG
	TaCht6	ATTGTGAGGAAGAAGGCGGA	TGCGAGGTCTGTTTCTCCTT
	TaCht7	CTCAGCGGGTACAAAGTTCG	GTGGTGCTTTCTCTCTCCCT
	TaCht8	ACCTTCACCTTAGCAGACCC	GGAACGGCCGATTCTTCATC
	TaCht10	CCGGAAATGGATTAGGTGCG	CCTTGTGGCGGATCATGAAG
	TaCht11	GGCTTTGGTTCTCTTGGCTC	CACTCCCTGGTAGTCATCCG
	TaCht-h	CGGCATACAATCAGCTCGTC	GGTATCTTCCGCCCTTCCTT
	TaIDGF	TCCATCTACTACGACGTGCC	CTTGCAGCCAGTAGTTGACG
	TaEF1α	CCTGGGCACAGAGATTTCAT	GATCAGCTGCTTGACACCAA
dsRNA synthesis	*TaCht1*	(T7)TCCCACTCAGAACAGAAG	(T7)ATACTCGGTCGCTACAAT
	TaCht2	(T7)TTTTGACCGTGGCTTCTG	(T7)TCCATCCATAACAGAAGC
	TaCht3	(T7)TAACTCTGGTGCCTATGT	(T7)GAAACTGTAGCCATCAAG
	TaCht5	(T7)TCCAAGTACTCCCACATG	(T7)CTCTGTCCACTTCTGAAC

	TaCht6	(T7)TTACAGACCTCCAAAGCG	(T7)TTCGGTGAACGACAAAGT
	TaCht7	(T7)TCAGCCAGCGTGAACAGA	(T7)GACCACCCACCAATAGCC
	TaCht8	(T7)GAACTTGGGCAACATACA	(T7)ACGAGACAGCAGCAGTAA
	TaCht10	(T7)GACTATGATCTCGTCCGAGGAG	(T7)GCATGATTGAACGTGAAGGCA
	TaCht11	(T7)ACTATCAATCCATCCCTAA	(T7)AAACCAAGTGAGCCAAGA
	TaCht-h	(T7)GTTGGTGCTTACTTCGTT	(T7)CACTACCTGAATCTTATCCC
	TaIDGF	(T7)CGGAGGTGATGCCGATAA	(T7)GATGCCTTCAGTCTTGGTGTA
	GFP	(T7)GTGGAGAGGGTGAAGG	(T7)GGGCAGATTGTGTGGAC

*T7, T7 RNA polymerase recognition sequence (TAATACGACTCACTATAGGG).
