# Supplementary material for: Identification and Functional Characterization of Chitinase Genes During Larva–Pupa–Adult Transitions in Tuta absoluta
Source: Insects. 2026 Jan 20;17(1):114. doi: 10.3390/insects17010114 (PMC12841776; doi:10.3390/insects17010114)
Supplement: Supplementary file 1 [file insects-17-00114-s001.zip › Table S3.pdf]

Table S3. Scientific names of chitinase sequences from different insect species used in constructing the phylogenetic tree.

Gene name	GenBank accession number	Insect Species
TaCht1	PX849541	
TaCht2	PX849542	
TaCht3	PX849543	
TaCht5	PX849544	
TaCht6	PX849545	
TaCht7	PX849546	*Tuta absoluta*
TaCht8	PX849547	
TaCht10	PX849548	
TaCht11	PX849549	
TaCht-h	PX849550	
TaIDGF	PX849551	
AgCht5-1	AEE44123.1	
AgCht5-2	AEE44124.1	
AgCht5-3	AEE44125.1	
AgCht5-4	AEE44126.1	
AgCht5-5	AEE44127.1	
AgCht7	XP_308858.4	*Anopheles gambiae*
AgCht8	XP_316448.2	
AgCht11	XP_310662.4	
AgCht13	XP_314312.4	
AgCht16	XP_319801.4	
AgIDGF	XP_001237925.1	
BmCht1	XP_004931749.1	
BmCht2	XP_004933352.2	
BmCht3	XP_012551241.1	
BmCht5	AAB47538.1	
BmCht6	XP_012553393.2	*Bombyx mori*
BmCht7	XP_004922005.1	
BmCht11	XP_004926923.1	
BmCht-h	BAC67246.1	
BmIDGF	NP_001036847	
CqCht4	XP_001841680.1	
CqCht5-1	XP_001863384.1	
CqCht5-2	XP_001863385.1	
CqCht6	XP_001862401.1	
CqCht7	XP_001862994.1	*Culex quinquefasciatus*
CqCht8	XP_001841679.1	
CqCht10	XP_001857861.1	
CqCht11	XP_001868126.1	
CqCht13	XP_001845767.1	
CqCht15	XP_001841677.1	
DmCht1	NP_609190	*Drosophila melanogaster*
DmCht4	NP_524962	

DmCht7	NP_647768	
DmCht9	NP_611543	
DmCht10	EAA46011	
DmCht11	NP_572361	
DmIDGF1	NP_477258	
DmIDGF2	NP_477257	
DmIDGF3	NP_723967	
DpCht-h	EHJ71822	*Danaus plexippus*
NlCht3	AJO25038.1	*Nilaparvata lugens*
NlCht5	AJO25040.1	
NlCht10	AJO25045.1	
NlIDGF	AJO25056.1	
PoCht2	KAL5638095.1	*Phthorimaea operculella*
PoCht3	KAL5652070.1	
PoCht5	KAL5633401.1	
PoCht6	KAL5637311.1	
PoCht7	KAL5635430.1	
PoCht8-1	KAL5644636.1	
PoCht8-2	KAL5644634.1	
PoCht8-3	KAL5644635.1	
PoCht10	KAL5633063.1	
PoCht11	KAL5633245.1	
PoIDGF	KAL5647016.1	
PxCht1	AZS52288.1	*Plutella xylostella*
PxCht2	AZS52289.1	
PxCht3	AZS52290.1	
PxCht5	AZS52291.1	
PxCht6-1	AZS52292.1	
PxCht6-2	AZS52293.1	
PxCht7	AZS52294.1	
PxCht8	AZS52295.1	
PxCht10	AZS52296.1	
PxIDGF	*AZS52300.1*	
OfCht-h	BAE16587	*Ostrinia furnacalis*
SlCht-h	AGW23592	*Spodoptera litura*
TcCht2	XP_970191	*Tribolium castaneum*
TcCht3	EFA08056	
TcCht4	NP_001073567	
TcCht5	NP_001034524	
TcCht6	XP_967813	
TcCht7	NP_001036035	
TcCht8	NP_001038094	
TcCht9	NP_001038096	
TcCht10	NP_001036067	
TcCht11	XP_015836414.1	
TcIDGF2	NP_001038092	
