# Supplementary material for: Identification and Functional Characterization of Chitinase Genes During Larva–Pupa–Adult Transitions in Tuta absoluta
Source: Insects. 2026 Jan 20;17(1):114. doi: 10.3390/insects17010114 (PMC12841776; doi:10.3390/insects17010114)
Supplement: Supplementary file 1 [file insects-17-00114-s001.zip › Table S4.pdf]

**Table S4. Developmental and tissue-specific expression profiles of eleven *TaCht* genes in *Tuta absoluta*.**

**(1) Developmental distribution expression profiles.**

| | *TaCht1* | *TaCht2* | *TaCht3* | *TaCht5* | *TaCht6* | *TaCht7* | *TaCht8* | *TaCht10* | *TaCht11* | *TaCht-h* | *TaIDGF* |
|---|---|---|---|---|---|---|---|---|---|---|---|
| **1L** | 1.01±0.10 | 1.77±0.16 | 1.55±0.26 | 1.77±0.16 | 3.98±0.57 | 13.74±0.36 | 3.54±0.08 | 3.15±0.32 | 4.00±0.20 | 1.77±0.16 | 1.77±0.16 |
| **2L** | 1.88±0.07 | 1.00±0.04 | 1.46±0.15 | 1.00±0.04 | 16.91±1.70 | 3.04±0.22 | 1.74±0.07 | 2.71±0.05 | 5.53±0.53 | 1.00±0.04 | 1.00±0.04 |
| **3L** | 2.36±0.30 | 1.50±0.15 | 1.09±0.11 | 1.50±0.15 | 10.31±0.91 | 1.00±0.04 | 1.19±0.09 | 1.02±0.11 | 1.01±0.09 | 1.50±0.15 | 1.50±0.15 |
| **4L** | 4.27±0.60 | 2.23±0.19 | 0.83±0.05 | 2.23±0.19 | 5.53±0.79 | 0.64±0.05 | 2.46±0.47 | 1.37±0.10 | 1.69±0.20 | 2.23±0.19 | 2.23±0.19 |
| **PP** | 2.81±0.36 | 3.75±0.42 | 9.69±0.54 | 3.75±0.42 | 3.50±0.47 | 1.53±0.17 | 2.26±0.22 | 6.98±0.55 | 2.45±0.09 | 3.75±0.42 | 3.75±0.42 |
| **P1** | 6.33±0.60 | 4.58±0.69 | 1.02±0.12 | 4.58±0.39 | 3.16±0.43 | 2.42±0.31 | 1.05±0.17 | 1.97±0.20 | 10.49±0.13 | 4.58±0.69 | 4.58±0.69 |
| **P2** | 7.99±0.53 | 3.44±0.15 | 1.00±0.05 | 3.44±0.15 | 2.58±0.22 | 9.61±0.31 | 1.90±0.15 | 1.74±0.15 | 6.87±0.69 | 3.44±0.15 | 3.44±0.15 |
| **P3** | 17.13±1.34 | 6.45±0.32 | 1.16±0.20 | 6.45±0.68 | 9.83±2.00 | 7.53±0.78 | 2.77±0.31 | 5.8±0.39 | 5.13±0.47 | 6.45±0.68 | 6.45±0.68 |
| **P4** | 9.12±0.68 | 11.48±0.47 | 21.05±1.85 | 11.48±0.47 | 19.64±0.46 | 4.31±0.31 | 1.60±0.19 | 1.93±0.14 | 6.41±0.51 | 11.48±0.95 | 10.98±0.5 |
| **P5** | 6.15±0.59 | 6.85±0.57 | 51.71±4.26 | 6.85±0.57 | 191.75±2.94 | 42.77±3.78 | 6.13±0.43 | 7.85±0.54 | 7.17±0.51 | 6.85±0.57 | 6.85±0.57 |
| **P6** | 14.03±0.53 | 6.54±0.56 | 80.53±4.18 | 6.54±0.56 | 78.13±5.15 | 53.07±3.17 | 15.63±1.27 | 1.52±0.05 | 7.11±0.54 | 6.54±0.56 | 6.54±0.56 |
| **P7** | 6.99±0.09 | 5.73±0.30 | 68.27±8.23 | 5.73±0.30 | 16.41±1.04 | 9.24±0.53 | 9.73±0.90 | 2.42±0.26 | 5.29±0.58 | 5.73±0.30 | 5.73±0.30 |
| **A1** | 222.10±13.50 | 14.78±0.53 | 4.54±0.37 | 14.78±0.40 | 2.20±0.01 | 20.06±0.83 | 38.27±4.08 | 8.04±0.19 | 8.34±0.49 | 14.78±0.40 | 26.18±2.56 |

| | *TaCht1* | *TaCht2* | *TaCht3* | *TaCht5* | *TaCht6* | *TaCht7* | *TaCht8* | *TaCht10* | *TaCht11* | *TaCht-h* | *TaIDGF* |
|---|---|---|---|---|---|---|---|---|---|---|---|
| **A2** | 42.12±4.49 | 2.87±0.24 | 1.15±0.07 | 2.87±0.24 | 1.08±0.07 | 14.16±0.32 | 27.26±2.31 | 6.41±0.15 | 3.68±0.30 | 2.87±0.24 | 32.76±2.57 |
| **A3** | 81.08±1.79 | 9.98±0.39 | 2.43±0.21 | 9.98±0.39 | 1.57±0.13 | 33.23±1.76 | 112.65±9.98 | 1.06±0.06 | 11.77±0.43 | 9.98±0.39 | 45.76±3.76 |
| **A4** | 53.93±2.69 | 3.86±0.14 | 1.94±0.06 | 3.86±0.14 | 1.60±0.17 | 13.11±0.55 | 70.16±2.45 | 4.67±0.43 | 6.06±0.38 | 3.86±0.14 | 72.52±4.71 |
| **A5** | 62.93±6.62 | 4.53±0.47 | 1.24±0.21 | 4.53±0.47 | 0.98±0.20 | 11.48±0.69 | 49.73±3.84 | 9.97±0.35 | 5.25±0.38 | 4.53±0.63 | 46.54±7.15 |

**(2) Tissue distribution expression profiles.**

| | *TaCht1* | *TaCht2* | *TaCht3* | *TaCht5* | *TaCht6* | *TaCht7* | *TaCht8* | *TaCht10* | *TaCht11* | *TaCht-h* | *TaIDGF* |
|---|---|---|---|---|---|---|---|---|---|---|---|
| **HD** | 1.00±0.04 | 3.64±0.08 | 5.09±0.17 | 11.61±0.17 | 14.90±1.40 | 9.13±0.40 | 1.34±0.13 | 17.01±0.58 | 1.02±0.12 | 6.17±0.25 | 1.01±0.07 |
| **IN** | 24.04±1.42 | 58.94±4.25 | 10.47±0.31 | 198.54±11.32 | 105.52±4.97 | 142.55±9.90 | 10.39±0.50 | 56.82±2.17 | 44.14±2.94 | 369.29±31.09 | 47.05±4.27 |
| **FB** | 3.94±0.08 | 1.51±0.14 | 1.00±0.05 | 1.00±0.01 | 3.18±0.13 | 3.28±0.17 | 3.02±0.19 | 3.73±0.17 | 7.85±0.59 | 1.69±0.21 | 5.93±0.40 |
| **MT** | 11.35±1.41 | 10.66±0.97 | 6.09±0.48 | 43.68±4.05 | 28.31±2.16 | 39.55±4.16 | 88.16±11.45 | 48.11±4.01 | 36.69±2.64 | 335.95±24.19 | 54.83±4.73 |
| **FG** | 18.32±1.69 | 6.21±0.52 | 5.01±0.55 | 15.25±1.09 | 20.22±1.21 | 10.84±0.64 | 354.19±15.41 | 24.66±3.11 | 197.21±11.56 | 44.35±7.22 | 20.11±1.15 |
| **MG** | 10.32±0.70 | 1.54±0.18 | 1.74±0.02 | 4.26±0.35 | 8.78±0.32 | 1.14±0.04 | 243.13±18.17 | 5.74±0.41 | 242.40±11.75 | 18.52±1.27 | 10.55±0.85 |

Note: The three independent replicates of group were presented as mean±SE.
